# Long non-coding RNA MT1DP shunts the cellular defense to cytotoxicity through crosstalk with MT1H and RhoC in cadmium stress

**DOI:** 10.1038/s41421-017-0005-y

**Published:** 2018-01-30

**Authors:** Ming Gao, Minjun Chen, Changying Li, Ming Xu, Yun Liu, Min Cong, Nan Sang, Sijin Liu

**Affiliations:** 1 State Key Laboratory of Environmental Chemistry and Ecotoxicology, Research Center for Eco-Environmental Sciences, Chinese Academy of Sciences, Beijing, 100085 China; 2 University of Chinese Academy of Sciences, Beijing, 100049 China; 3 College of Environment and Resource, Research Center of Environment and Health, Shanxi University, Taiyuan, Shanxi 030006 China; 4 Liver Research Center, Beijing Friendship Hospital, Capital Medical University, Beijing, 100050 China; 5 Key Laboratory of Ion Beam Bioengineering, Hefei Institutes of Physical Science, Chinese Academy of Sciences and Anhui Province, Hefei, Anhui 230031 China

## Abstract

Metallothioneins (MTs) are known to protect cells against oxidative stress, especially providing protection against cadmium (Cd) toxicity in hepatocytes. There are various gene variants and pseudogenes for MTs; however, there is little understanding on the functions of those non-coding MT members that are known to be expressed as long non-coding RNAs (lncRNAs) nowadays. Different from most protein-coding MT members, MT1DP was here found that remarkably induced to provoke cytotoxicity in hepatocytes in response to Cd treatment. MT1DP exerted such a pro-apoptotic function in Cd-treated hepatocytes through interacting with two partners: RhoC and MT1H. On one hand, MT1DP interacted with RhoC protein to increase the latter’s stability by preventing lysosome-dependent protein degradation. Therefore, upon Cd stress, MT1DP/RhoC complex was quickly reinforced to activate RhoC-CCN1/2-AKT signaling and potentiate Ca^2+^ influx, leading to enhanced Cd uptake and elevated Cd toxicity. On the other hand, MT1H, a protein-coding member of the MT family with little known function, was found to quickly respond to Cd exposure along with MT1DP. Mechanistically, MT1H and MT1DP were uncovered to mutually protect each other through a reciprocal ceRNA mechanism, building up a positive feedback loop to enforce MT1DP-conducted signaling upon Cd exposure. Moreover, MT1DP was found to contribute much more to the activation of RhoC-CCN1/2-AKT signaling than MT1H. Considered together, we here unveiled a mystery whether a pseudogene within the MT family, MT1DP, has actual biological functions in regulating Cd-induced cellular defense. Our findings unearthed an important role of pseudogene MT1DP in calibrating the cellular machinery to switch the cellular defense to cytotoxicity through crosslinking an interplay between its two partners, namely MT1H and RhoC, under cadmium stress.

## Introduction

Mammals have developed evolutionarily conserved intricate defense mechanisms against stress in response to toxic substances, such as antioxidant agents, detoxification enzymes, pro-survival signaling, autophagy and metal-binding proteins^1–4^. Thus far, metallothioneins (MTs) have been extensively investigated for their protection from cadmium (Cd) toxicity^5, 6^. The pivotal role of MTs in Cd detoxification has been established prominently due to Cd sequestration through their high-affinity binding inside cells, resulting in reduced Cd mass to prevent damage to cellular organelles^5, 6^. This mechanism is further verified by MT-transgenic mice, as increased MT expression endowed mice with enhanced tolerance to Cd toxicity and MT-null mice were contrastively more vulnerable^7, 8^. There are at least 16 members in the MT family including 12 protein-coding variants (i.e., MT1A, MT1B, MT1E, MT1F, MT1G, MT1H, MT1L, MT1M, MT1X, MT2, MT3 and MT4) and 4 pseudogenes (namely MT1CP, MT1DP, MT1IP, and MT1JP) without protein-coding functionality in the human genome. Yet, the inter-regulation among these members (e.g., synergism and antagonism) and particularly their biological functions of non-coding pseudogenes (now known as long non-coding RNAs, lncRNAs) are still elusive.

As a subtype of non-coding RNAs, lncRNAs are transcribed in sense or antisense to protein-coding genes, with the length >200 nucleotides^9, 10^. Accumulating studies have documented the necessary contribution of lncRNAs to fundamental physiological homeostasis and versatile biological functions, whereas deregulated expression for certain lncRNAs may cause diverse pathologies including cancers and diabetes^11–13^. LncRNAs implement their functions through modulation of gene expression (such as chromatin remodeling and posttranscriptional modification of target mRNAs) and regulation of protein activity via physical interaction^14, 15^. Historically, pseudogenes were defined as “junk” DNA, such as pseudogenes in the MT family, due to their lack of protein-coding functionality, whereas recent reports suggested that many pseudogenes express non-coding RNAs, including lncRNAs^16^. However, the biological significance of most non-coding RNAs transcribed from pseudogenes is largely unknown^16, 17^.

Given that the pseudogenes in the MT family themselves are lncRNAs, we postulated that these lncRNAs may antagonize or synergize the real MTs at the transcriptional or posttranscriptional level in order to orchestrate cellular defense and cytotoxicity. In the current study, one of the pseudogenes, MT1DP, was found to be dramatically induced in hepatocytes by Cd treatment. Different from MT1/2, MT1DP is present in human genome but not in mouse genome^18^. It was reported that MT1DP acted as a tumor suppressor through negatively regulating YAP and Runx2 to promote apoptosis of liver cancer cells^19^; however, the biological functions of MT1DP are still almost unexploited thus far, with no clue linking to Cd toxicity. Our results unearthed a vital role of lncRNA MT1DP in antagonizing the cytoprotective role of most MT members to enhance Cd-induced toxicity in hepatocytes through physically interacting with RhoC (Ras homolog gene family, member C). Stabilized MT1DP/RhoC complex thereafter activated CCN1/2-AKT signaling to promote cell death dependent on elevated Cd uptake. Meanwhile, the function of MT1DP was further enhanced by a MT family member, MT1H, which functioned as a competing endogenous RNA (ceRNA) to block miR-214-conducted suppression on MT1DP. These findings together unveiled the molecular bases underlying MT-associated cytotoxicity versus cellular defense under Cd stress. This study would open a path to understand the regulation of a pseudogene in a form of lncRNA on its according protein-coding gene.

## Results

### Differentially stimulated expression of the MT1 family members by Cd in hepatocytes

**Fig. 1:**
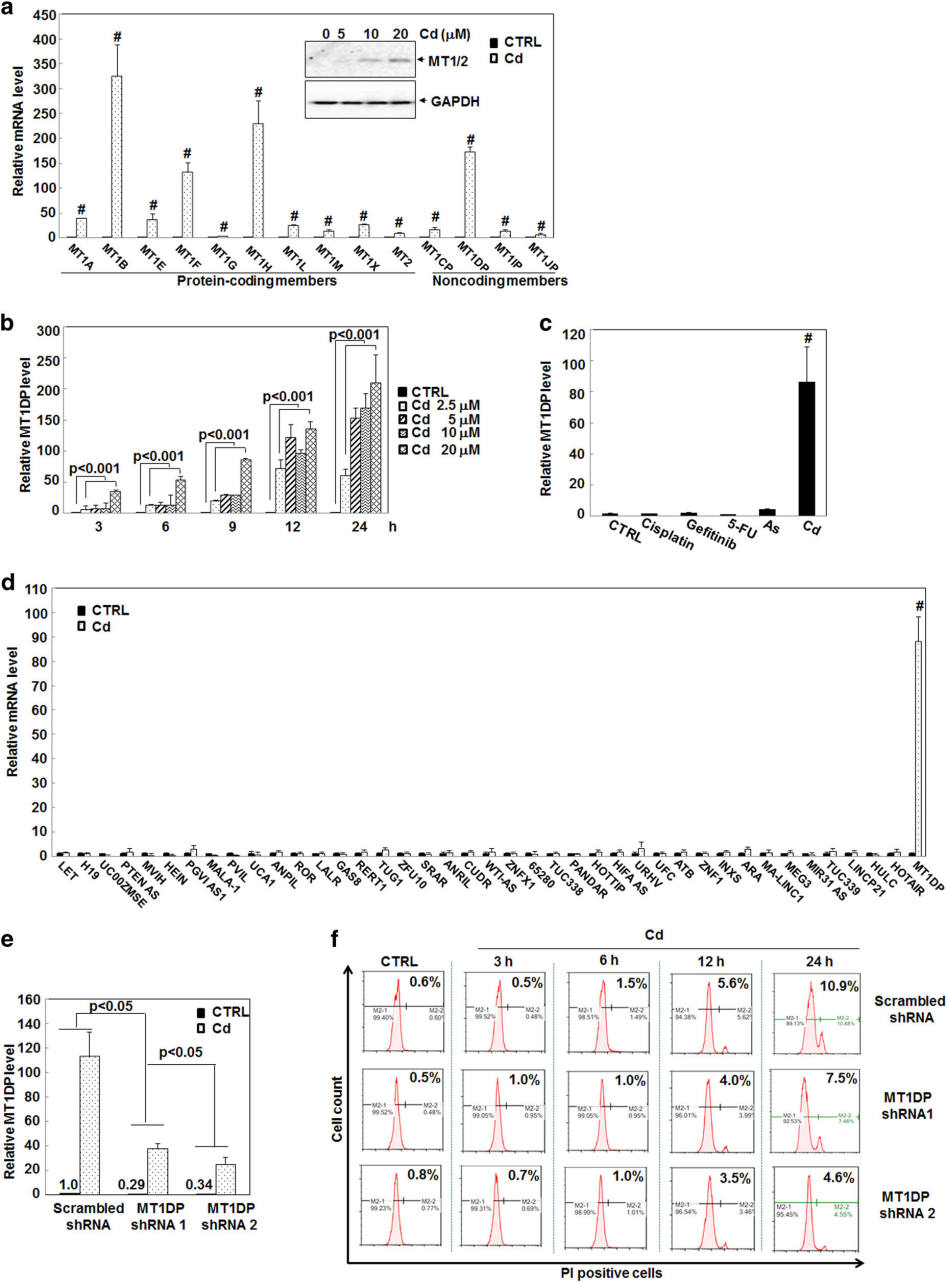
MT1DP induction promotes Cd-induced cell death. **a** Relative expression levels of MT members in HepG2 cells treated with Cd at 20 μmol/L for 24 h. Inserted panel, western blot analysis of MT1/2 proteins in HepG2 cells treated with Cd for 24 h. **b** MT1DP levels upon exposure to Cd at various concentrations over time determined by qRT-PCR (*n* = 3). **c** Relative expression levels of MT1DP in HepG2 cells upon treatment with 2 μg/mL cisplatin, 5 μM/mL gefitinib, 50 μg/mL 5-FU, 10 μmol/L arsenic (As), and 20 μmol/L Cd through qRT-PCR assay (*n* = 3). **d** Expression changes of lncRNAs in HepG2 cells exposed to 10 μmol/L Cd for 24 h, as characterized by qRT-PCR analysis (*n* = 3). **e** Relative expression levels of MT1DP in MT1DP^low^ cells. The endogenous MT1DP was stably knocked down in HepG2 cells, as evidenced by qRT-PCR assay (*n* = 3), with relative expression levels labeled above the bars for MT1DP-shRNA #1 and MT1DP-shRNA #2 transfectants. Expression changes of MT1DP in cells responded to Cd at 20 μmol/L were also detected by qRT-PCR (*n* = 3). **f** The proportions of PI-positive in scrambled-shRNA control cells and MT1DP-low cells in response to 20 μmol/L Cd for 3, 6, 12 and 24 h, determined by flow cytometry analysis (*n* = 3)

To shed light on the interplay among the MT family members, we first compared the expression profiles of 10 real MT1/2 genes and 4 pseudogenes in hepatocytes upon Cd treatment. As shown in Fig. 1a, the 10 protein-coding members were markedly stimulated in HepG2 cells by Cd at 20 μM, analogous to previous studies^20, 21^. The induction of protein-coding members was confirmed at the protein levels, as evidenced by the western blot results (Fig. 1a), supporting of a critical role of MT1/2 against Cd toxicity^6^. Moreover, all non-coding members, MT1CP, MT1DP, MT1IP, and MT1JP were also induced by Cd, especially for MT1DP with >100-fold increase (Fig. 1a, *P* < 0.001). Of note, the increase of MT1DP expression was the greatest with about 10-fold induction relative to other non-coding members (Fig. 1a, *P* < 0.001). This finding thus implied a potential role of MT1DP in modulating Cd-induced cell responses.

### MT1DP selectively responded to Cd stress and enhanced Cd-induced cell death

To elucidate the biological function of MT1DP under Cd stress, MT1DP responsiveness was determined in hepatocytes in response to Cd at various concentrations over the time course. Overall, a dose- and time-dependent induction of MT1DP expression was found in HepG2 cells with the concentrations ranging from 2.5 to 20 μM at 3, 6, 9, 12, and 24 h (Fig. 1b, *P* < 0.001). A quick response of MT1DP induction was observed early at 3 h, and a greater increase of MT1DP level was found over time, with >200-fold increase in cells treated with 20 μM Cd at 24 h (Fig. 1b, *P* < 0.001). MT1DP was also greatly induced upon even lower concentrations of Cd exposure (Fig. S1, *P* < 0.001). These observations indicated that the expression of MT1DP quickly and robustly responded to Cd-mediated stress. Furthermore, the selectivity of MT1DP induction by Cd was examined using a few agents that are able to provoke cytotoxicity including cisplatin, gefitinib, 5F-dUMP (5-FU), and arsenic. We deliberately used the bespoke concentrations for these regents at which they incurred comparable toxicities to cells with approximately 10% cell death at 24 h (Fig. S2, *P* > 0.05). In the meantime, cisplatin, gefitinib, 5-FU, and arsenic elicited rather weak responsiveness of MT1DP induction with the greatest increase in arsenic-treated cells (an increase of 4-fold) relative to untreated cells, whereas a >80-fold increase was observed in Cd-treated cells (Fig. 1c, *P* < 0.001), suggesting a selective responsiveness of MT1DP induction to Cd. Afterward, the selectivity of MT1DP for Cd was interrogated through assessing the expression profile of other 40 lncRNAs that were reported to be involved in hepatic metabolism and stress responses^22–25^. As shown in Fig. 1d, the expression of all these lncRNAs were not significantly altered or only slightly induced in HepG2 cells by Cd at 10 μM, whereas MT1DP was considerably induced by nearly 100-fold (*P* < 0.001). These results collectively signified an exclusive involvement of MT1DP in response to Cd treatment, and also implied an important role of MT1DP in modulating Cd-induced cellular responses.

Since MT1DP has seldom been studied thus far, we further shed light on its function in Cd stress through knocking down the endogenous MT1DP level and overexpression of an exotic MT1DP construct. As a result of viral vector-based short hairpin RNA (shRNA) infection, the endogenous MT1DP expression was greatly reduced by approximately 70% in two shRNA transfectants (MT1DP-shRNA1 and 2), here named MT1DP^low^ cells (Fig. 1e, *P* < 0.05). Nevertheless, the expression level was still >60% lower in MT1DP^low^ cells relative to wild-type (WT) cells upon Cd at 20 μM (Fig. 1e, *P* < 0.05). Afterward, cell death was evaluated in MT1DP^low^ cells and WT cells with or without Cd treatment. As shown in Fig. 1f, MT1DP reduction did not affect cell death in cells without Cd treatment, whereas Cd-induced cell death was greatly repressed in MT1DP^low^ cells over the time course relative to WT cells (*P* < 0.005), suggesting a cell death promotion role of MT1DP under Cd toxicity. When comparing the timing of MT1DP induction and the occurrence of cell death, MT1DP induction responded quickly as early as 3 h post Cd exposure (Fig. 1b), whereas a significant inhibition of cell death in MT1DP^low^ cells was recognized at 24 h (Fig. 1f), indicative of chronological regulation of cellular defense and cytotoxicity by MT1DP. To substantiate this finding, we elevated the cellular MT1DP level in HepG2 cells (here called MT1DP^high^ cells) through overexpression of a constructed MT1DP plasmid (Fig. S3A, *P* < 0.001). Conversely, cell death was largely enhanced in MT1DP^high^ cells by 2.4% and 8.5% upon 10 and 20 μM Cd for 24 h, compared with WT cells, respectively (Fig. S3B, *P* < 0.05). Moreover, to corroborate the contribution of MT1DP to Cd-induced cell death, we further looked into cell death incurred by cisplatin, gefitinib, 5-FU arsenic, and Cd. As shown in Fig. S2, no significant difference was identified in MT1DP^low^ cells compared with WT cells responding to cisplatin, gefitinib, 5-FU, and arsenic except for Cd at the concentrations with comparable cytotoxicity (*P* > 0.05). Together, these data uncovered that increased MT1DP chronologically promoted cell death of hepatocytes upon Cd, and pointed out MT1DP as a selective responsive regulator of cell death in Cd stress.

### MT1DP interacts with RhoC to promote Cd-induced cell death

**Fig. 2:**
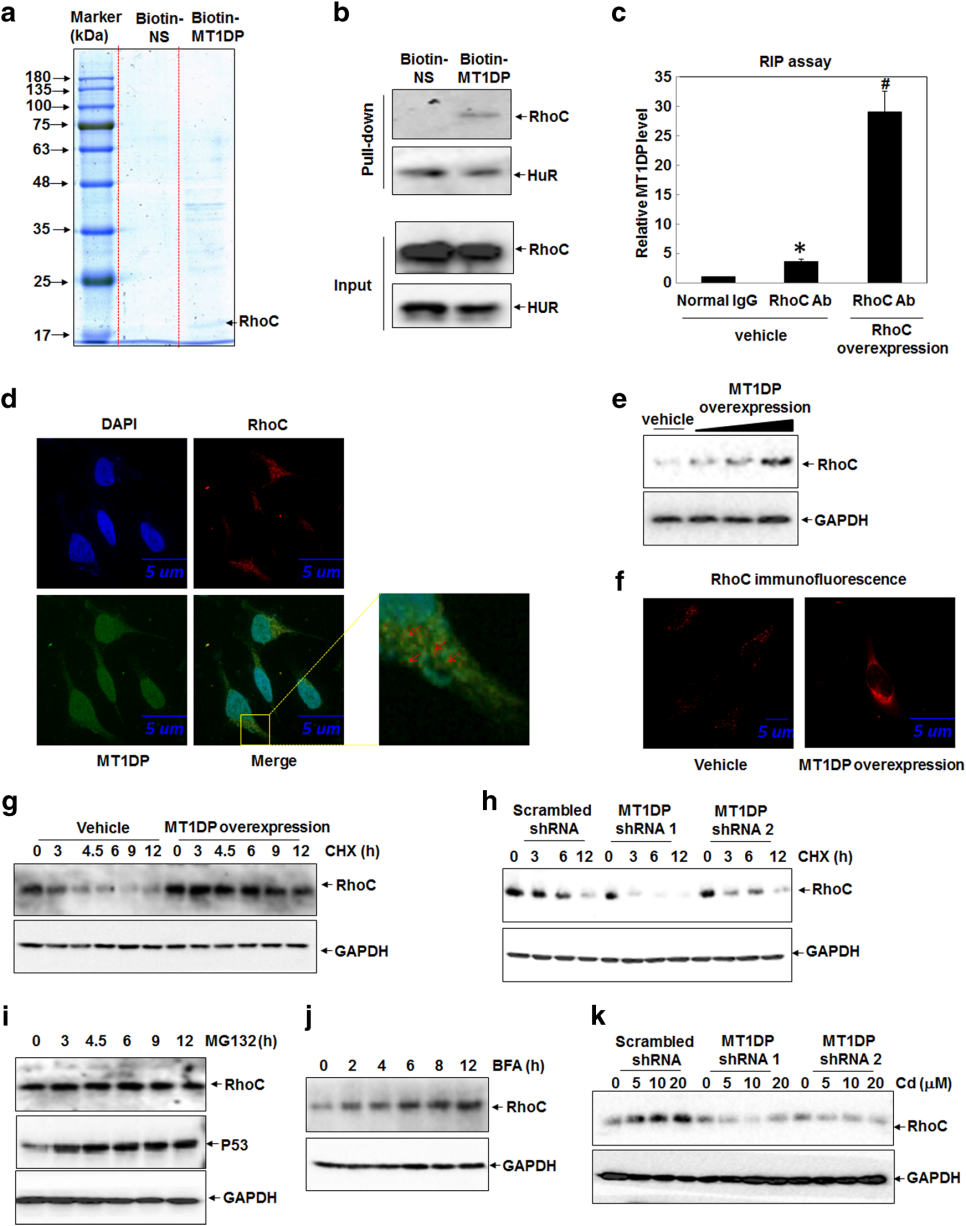
MT1DP interacts with RhoC and increases its protein stability to reinforce cell death upon Cd treatment. **a** MT1DP partners were surveyed by RNA-pull-down assay and mass spectrometry. **b** Western blot analysis of RhoC protein concentrations in biotin-MT1DP and biotin-NS pull-down complexes. NS denotes non-sense RNA probes. **c** RIP assay was performed to determine the binding of RhoC with MT1DP RNA. The enrichment of MT1DP RNA was measured by qRT-PCR assay in normal IgG pull-down complex from vehicle control cells and anti-RhoC Ab pull-down complexes from vehicle control and RhoC overexpression cells, respectively (*n* = 3). **d** The fluorescence of RhoC (in red) and MT1DP (in green) was visualized by confocal microscopy. The red arrows indicate the overlapping (shown in yellow) between RhoC protein fluorescence and MT1DP RNA fluorescence. Nuclei are shown in blue through staining with DAPI. **e** HepG2 cells were transfected with increasing concentrations of MT1DP overexpression constructs for 24 h, and thereafter the protein contents of RhoC were determined by western blotting. **f** RhoC protein fluorescence (shown in red) was detected by confocal microscopy in vehicle control and MT1DP overexpression cells. **g** Western blot analysis of the protein levels of RhoC in vector control and MT1DP overexpression cells in response to 40 μmol/L CHX over the time course. **h** RhoC protein concentrations in scrambled control and MT1DP^low^ cells treated with 40 μmol/L CHX over time, detected by western blot analysis. HepG2 cells were treated with 20 μmol/L MG132 **i** and 10 nmol/L BFA **j** over time, and then the protein levels of RhoC and p53 were determined by western blot. **k** The protein content of RhoC in scrambled control and MT1DP^low^ cells in response to Cd for 6 h, determined by western blot analysis

Next, we embarked on the molecular basis underlying MT1DP-enhanced Cd toxicity by searching for the partners of MT1DP. Previous studies have demonstrated that lncRNAs exert their biological functions through diverse mechanisms including physical interactions with target proteins^15, 26, 27^. Therefore, we performed RNA pull-down assay using biotin-labeled MT1DP as the bait to look for its protein partners. As shown in Fig. 2a, biotin-labeled MT1DP pulled down much more proteins compared with biotin-labeled non-sense (NS) RNA with a similar length to MT1DP. Pulled-down proteins were thereafter subjected to mass spectrometry (MS) analysis. Of these identified proteins (Table S2), a number of small GTPase members and binding proteins were identified, such as RhoC, RhoG, Rab-4B, and Rab-7a, of which RhoC pronouncedly showed up due to its crucial role in governing cell proliferation, survival, and migration^28–30^, we here selected RhoC for further investigation. In support of the MS analysis, western blotting analysis confirmed the pull-down of RhoC protein by biotin-labeled MT1DP, whereas almost no RhoC could be visualized in the pull-down lysate by the NS probe (Fig. 2b). Here, RNA-binding protein human antigen R (HuR) was used as a loading control for the RNA pull-down assay (Fig. 2b).

To validate the binding between MT1DP and RhoC in vivo (namely in situ interaction in cells), RNA-binding protein immunoprecipitation (RIP) was further carried out. As shown in Fig. 2c, MT1DP RNA mass was enriched by approximately four times in the RhoC antibody (Ab)-pull-down complex greater than that in normal IgG pull-down complex (*P* < 0.05), showing the physical interaction between RhoC protein and MT1DP RNA. Additionally, the RhoC Ab enriched more MT1DP RNA in RhoC overexpression cells compared with that in vehicle control cells (29-fold vs 4-fold, Fig. 2c, *P* < 0.001), further verifying an active association of RhoC protein with MT1DP RNA. Importantly, fluorescent in situ hybridization (FISH) assay was further employed to detect the in situ interaction between RhoC protein and MT1DP RNA. As shown in Fig. 2d, the in situ hybridization results displayed that RhoC protein was nearly distributed in the cytoplasm of HepG2 cells (shown in red immunofluorescence), whereas MT1DP RNA was distributed cross nuclei and cytoplasm of cells (as observed in green fluorescence). Strikingly, massive colocalization of RhoC fluorescence and MT1DP fluorescence were visualized in yellow (as denoted by arrows) in the cytoplasm (Fig. 2d), substantiating the in situ formation of RhoC protein and MT1DP RNA complex in the cytosol. We further investigated whether RhoC and MT1DP could affect their levels and their reciprocal recruitment. As shown in Fig. S4A, RhoC overexpression posed no effect on the expression of MT1DP. By contrast, MT1DP overexpression reversely led to an accumulation of RhoC protein, as characterized by western blotting and RhoC immunofluorescence intensity (Figs. 2e, f), implying that MT1DP RNA in fact contributed to stabilizing RhoC protein. As RhoC is active in exerting its biological functions only upon GTP binding^31–33^ a specific pull-down using agarose beads coated with Rhotekin-RBD, which interacted with GTP-bound RhoC was performed in cells with or without MT1DP overexpression. As shown in Fig. S4B, a more significant enriched GTP-bound RhoC protein was found in cells with MT1DP overexpression, demonstrating the close interplay between MT1DP RNA and activated RhoC.

To unveil the mechanism responsible for this regulation by MT1DP, the protein stability of RhoC was suspected to be responsible. Hence, cycloheximide (CHX), an inhibitor of protein translation, was used to block overall protein synthesis. As a consequence, the cellular RhoC level dramatically dropped over the time course, and the half-life of RhoC protein was calculated to be 3–4.5 h (Fig. S5), suggestive of the regulation of RhoC protein stability. To test this possibility, the protein level of RhoC was assessed in MT1DP^high^ and MT1DP^low^ cells. As shown in Fig. 2g, the half-life of RhoC extended to 9–12 h in MT1DP^high^ cells, and it reversely declined to be <3 h in MT1DP^low^ cells (Fig. 2h), indicating that MT1DP could effectively regulate the turnover of RhoC protein. As two main proteolytic systems are responsible for the degradation of intracellular proteins, namely the ubiquitin/proteasome system and the lysosomal system^34^ we therefore interrogated both systems that may contribute to the degradation of RhoC. As shown in Fig. 2i, proteasomal inhibitor MG132 did not affect RhoC protein content over time. Here, p53 was used as a positive control, as accumulated p53 was observed over time upon MG132. In contrast, lysosomal inhibitor Bafilomycin A1 (BFA) induced an increase of RhoC protein concentration from 2 to 12 h (Fig. 2j), demonstrating that the degradation of RhoC protein was ascribed to the lysosomal system but not the ubiquitin/proteasome system.

We furthermore elaborated their partnership under MT1DP-promoted Cd toxicity. Along with the induction of MT1DP level (Fig. 1b), the RhoC concentration was also elevated in HepG2 cells upon Cd treatment from 5 to 20 μM in a dose-dependent manner (Fig. 2k). However, the elevation of RhoC was abolished in MT1DP^low^ cells in response to Cd (Fig. 2k). These results revealed a positive regulation of MT1DP on RhoC protein content upon Cd exposure. Further quantitative PCR (qPCR) analysis showed that the mRNA level of RhoC was not significantly altered in both WT and MT1DP^low^ cells upon Cd treatment (Fig. S6A, *P* > 0.05), ruling out the modulation of MT1DP on the transcription or mRNA stability of RhoC. To figure out the biological function of RhoC to Cd toxicity, cell death was surveyed in cells with RhoC knockdown (Fig. S6B). As a consequence, Cd-induced cell death was also markedly attenuated by nearly 50% upon RhoC knockdown in comparison with WT cells at 24 h (Fig. S6B and C, *P* < 0.001). Reversely, RhoC overexpression (Fig. S6D) resulted in greater cell death by 10.5% in cells upon Cd treatment, relative to WT cells (Fig. S6E, *P* < 0.001). Together, these findings uncovered an important regulation of Cd-induced cell death by MT1DP/RhoC complex.

### CCN1 and CCN2 are the responsive molecules under MT1DP/RhoC complex responding to Cd stress

Despite the activation of MT1DP/RhoC complex by Cd, the downstream cell death executors remained unexplored. To this end, the gene expression profile in MT1DP^low^ cells upon Cd treatment was screened using RNA-Seq technology. Among the genes with expression changes greater than twofold in both untreated and Cd-treated MT1DP^low^ cells relative to scrambled control cells, 32 candidate genes were selected for further analysis owing to their close implication in cell death regulation (Fig. S7A). Further quantitative reverse transcriptase-PCR (qRT-PCR) results recognized the best correlation for CCN1 and CCN2 of those 32 genes to the RNA-Seq data (Table S3 and data not shown), implying the likelihood of CCN1 and CCN2 as the downstream targets of MT1DP.

**Fig. 3:**
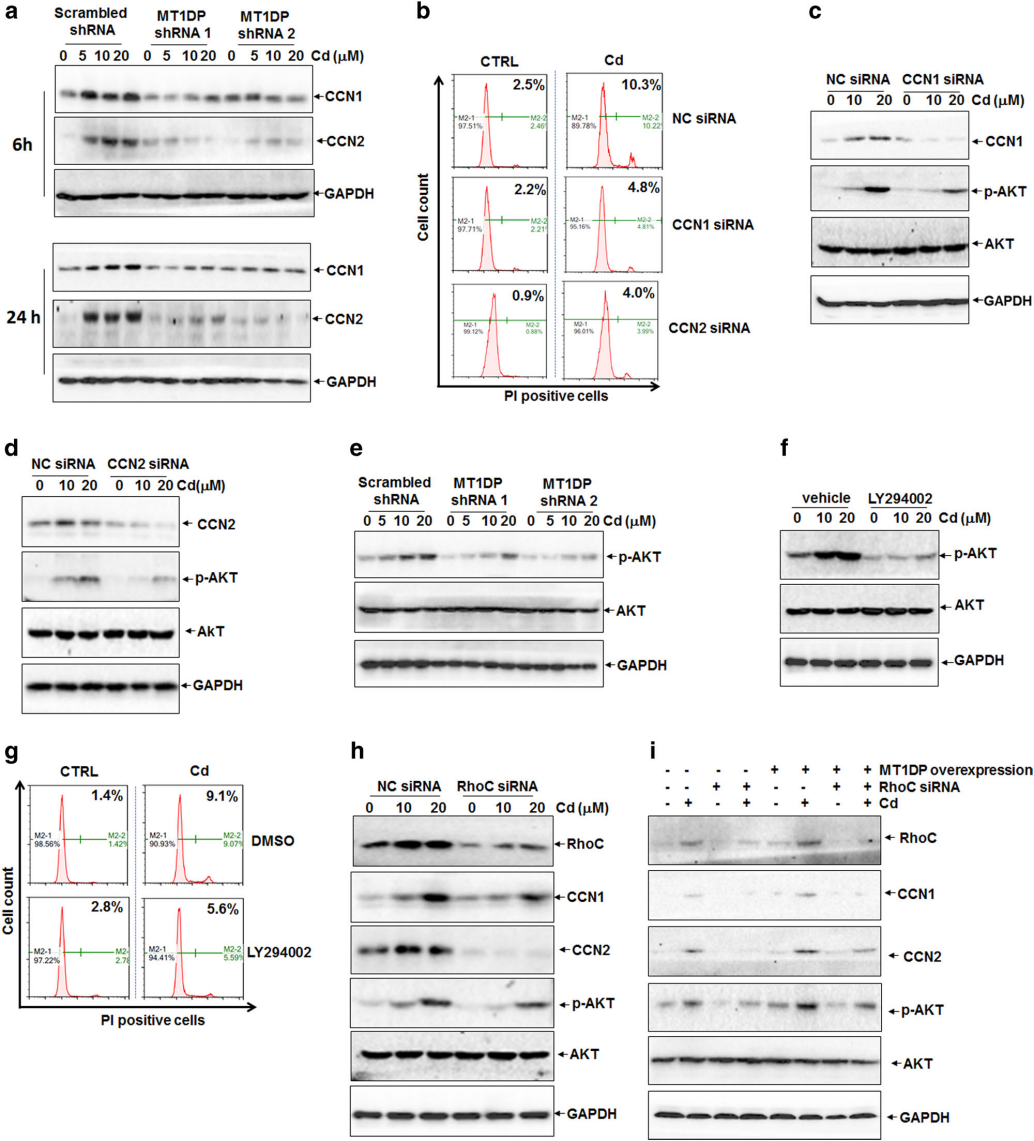
CCN1/2-AKT pathway is downstream of MT1DP/RhoC complex to reinforce Cd toxicity. **a** Western blot analysis of the protein concentrations of CCN1 and CCN2 in scrambled control and MT1DP^low^ HepG2 cells in response to Cd at indicated concentrations for 6 and 24 h. **b** Flow cytometry analysis of cell death with PI staining in cells after knocking down the endogenous CCN1 and CCN2 expression by specific siRNAs with or without Cd treatment at 20 μmol/L for 24 h. **c** Protein levels of CCN1 and phosphorylated AKT in NC-siRNA and CCN1-siRNA transfected cells in response to Cd. **d** CCN2 and phosphorylated AKT concentrations were compared by western blot assay in NC-siRNA and CCN2-siRNA transfected cells under Cd treatment. **e** Western blot analysis of phosphorylation of AKT in scrambled control and MT1DP^low^ cells under the treatment. **f**, **g** HepG2 cells were pretreated with a selective inhibitor against AKT (LY294002, 20 μmol/L) for 1 h, and then cells were treated with Cd, followed by determination of AKT phosphorylation through western blotting for 6-h Cd treatment **f** and cell death through flow cytometry analysis for 24-h Cd treatment. **h** Protein levels of RhoC, CCN1, CCN2 and phosphorylated AKT in NC-siRNA and RhoC-siRNA transfected cells upon Cd treatment for 6 h, as reflected by western blot analysis. **i** HepG2 cells were transfected with RhoC selective siRNA molecules for 24 h, followed by transfection of MT1DP overexpression constructs for another 24 h, and cells were then treated with 20 μmol/L Cd for 6 h. Finally, cells were collected for western blotting analysis of RhoC, CCN1, CCN2, phosphorylated AKT and total AKT concentrations

To address this likelihood, CCN1 and CCN2 levels were determined in MT1DP^low^ cells upon Cd treatment. As shown in Fig. S7B, Cd induced a remarkable increase of CCN1 and CCN2 at the mRNA level in scrambled control cells at 24 h (*P* < 0.05); however, this increase was greatly compromised by about 75% in MT1DP^low^ cells (*P* < 0.05). Analogously, CCN1 and CCN2 protein concentrations were upregulated in response to Cd in scrambled control cells at 6 and 24 h, and this increase of protein concentrations was also undermined in MT1DP^low^ cells (Fig. 3a). Reversely, overexpressed MT1DP led to approximately 2.5-fold elevation of CCN1 and CCN2 mRNA levels (Fig. S7C) and protein levels as well (Fig. S7D). To recognize the biological significance of CCN1 and CCN2 induction under Cd stress, cell death was assessed in cells with CCN1 and CCN2 knockdown (Fig. S7E). Consequently, Cd-induced cell death was suppressed by over 60% in CCN1- and CCN2-knockdown cells, compared with scrambled control cells (Fig. 3b, *P* < 0.001), indicative of a necessary role of CCN1 and CCN2 in conducting cell death downstream of MT1DP/RhoC complex under Cd stress.

### AKT signaling is the downstream target of CCN1 and CCN2 in response to Cd

Furthermore, we continued to look for the downstream signaling under CCN1 and CCN2 in response to Cd. Given the fact that previous studies have established the regulation of phosphoinositide-3-kinase (PI3K)-AKT signaling by CCN1 and CCN2^35, 36^ we thus hypothesized that CCN1 and CCN2 might enhance Cd-induced cell death through activating AKT signaling. To examine this hypothesis, AKT activation, namely AKT phosphorylation, was determined in cells upon Cd treatment. As shown in Figs. 3c, d, Cd at 10 and 20 μM, especially for the latter, greatly enhanced the phosphorylation of AKT along with the induction of CCN1 and CCN2. However, this activation of AKT was largely compromised in CCN1- and CCN2-knockdown cells upon Cd (Figs. 3c, d). To further depict this regulation, AKT phosphorylation was surveyed in MT1DP^low^ cells in comparison with scrambled control cells. As shown in Fig. 3e, dose-dependent AKT phosphorylation was significantly diminished in MT1DP^low^ cells upon to Cd, compared with scrambled control cells, signifying the regulation of AKT activation by MT1DP/RhoC-CCN1/2 signaling under Cd treatment.

Further, the biological role of AKT activation in Cd-induced cellular toxicity was investigated. As shown in Fig. 3f, a selective inhibitor LY294002 greatly repressed AKT phosphorylation in HepG2 cells upon Cd treatment at 10 and 20 μM, especially at 20 μM, compared with vehicle control cells. As a result, Cd-induced cell death was significantly reversed by about 30% by LY294002, relative to vehicle control (Fig. 3g, *P* < 0.05). Meanwhile, RhoC knockdown strategy was also used. Similar to RhoC inhibition results (Fig. 3h), RhoC knockdown greatly diminished AKT activation by Cd treatment at 6 and 24 h, compared with scrambled control cells (Fig. 3h and Fig. S8). In support of this finding, CCN1 and CCN2 concentrations were accordingly reduced in RhoC knockdown cells upon Cd treatment relative to scrambled control cells (Fig. 3h and Fig. S8). Additionally, Cd induced a marked increase of RhoC, CCN1, CCN2, and AKT phosphorylation in normal hepatocytes, L02 cells, and these inductions could be also significantly reversed upon MT1DP reduction (Fig. S9). Furthermore, we addressed whether RhoC was a critical executor in transducing the effect of MT1DP on the activation of CCN1/2-AKT pathway. As shown in Fig. S10, RhoC, CCN1, and CCN2 protein concentrations plus phosphorylated AKT level were all elevated in HepG2 cells in response to transfection of exotic MT1DP expression constructs for 48 h in comparison with control cells, and these elevations were reversed upon simultaneous RhoC knockdown, stressing the finding on the activation of CCN1-CCN2/AKT pathway by MT1DP through its partner RhoC. Moreover, MT1DP overexpression caused a greater increase of RhoC, CCN1, and CCN2 protein concentrations together with AKT phosphorylation under Cd treatment; however, these inductions were greatly reversed upon RhoC knockdown (Fig. 2i). These data thus signified the importance of MT1DP-RhoC-CCN1/2/AKT signaling cascade in response to Cd stress. Additionally, the other stress inducers, including cisplatin, gefitinib, 5-FU, and arsenic, minimally activated RhoC, CCN1, CCN2, and AKT relative to Cd (Fig. S11). Collectively, these results highlighted a crucial regulatory role of MT1DP/RhoC complex on AKT activation through CCN1/2 specifically responding to Cd-induced stress.

### AKT activation enhances calcium (Ca^2+^) influx and cellular Cd uptake

**Fig. 4:**
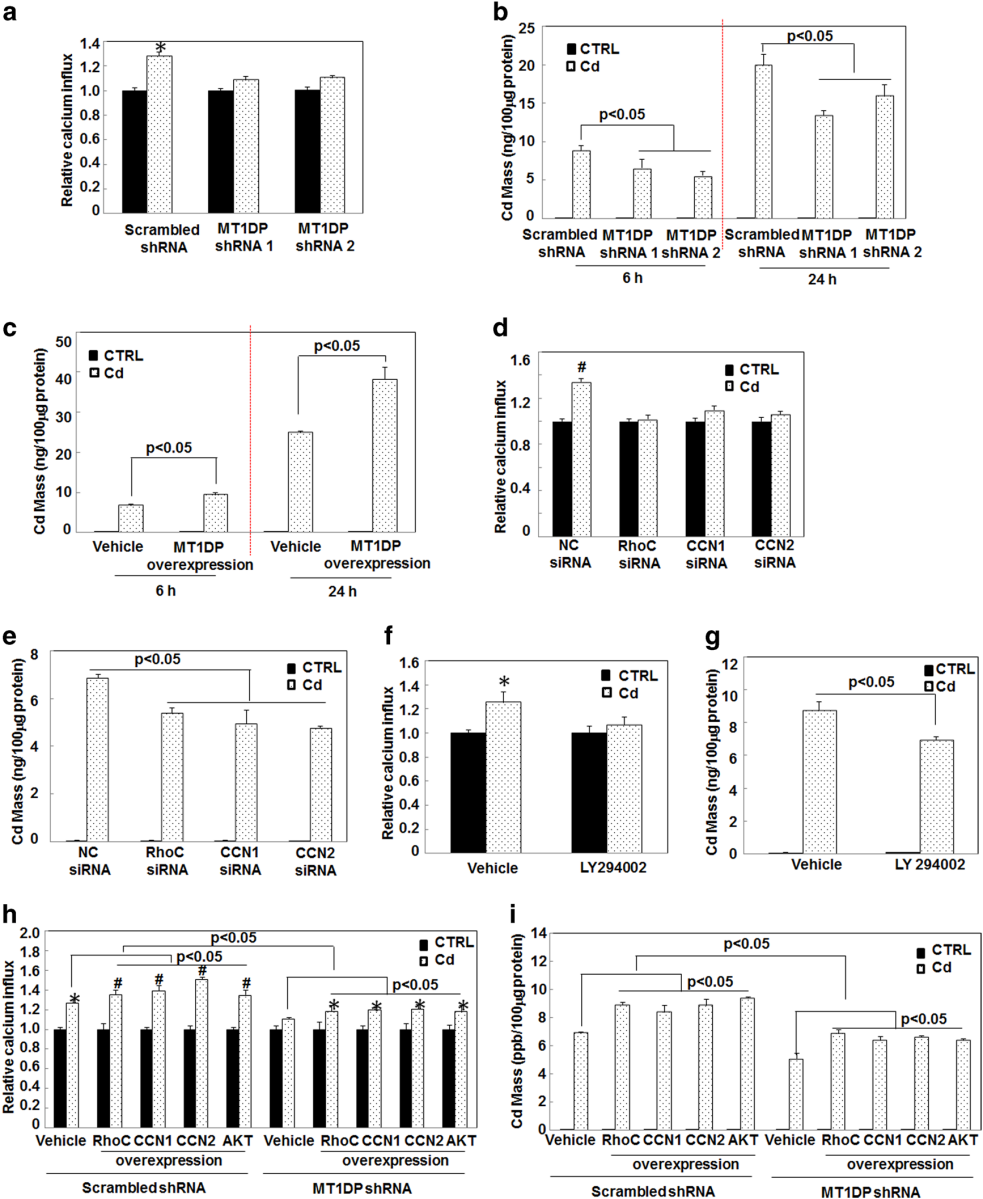
MT1DP/RhoC-CCN1/2-AKT pathway enforces Ca2+ influx and cellular uptake of Cd. **a** Scrambled control and MT1DP^low^ cells were pretreated with Cd at 20 μmol/L for 6 h, and then added with 5 μM Fluo-3AM for 1 h, followed by determination of cellular Ca^2+^ influx (*n* = 6). **b** Intracellular Cd mass in scrambled control and MT1DP^low^ cells in response to Cd at 20 μmol/L for 6 and 24 h, assayed by ICP-MS assay (*n* = 4). **c** Intracellular Cd content in vehicle control and MT1DP overexpression cells treated with Cd at 20 μmol/L for 6 and 24 h, measured by ICP-MS assay (*n* = 4). (**d**, **e**) Cellular Ca^2+^ influx **d** and mass **e** in NC-siRNA, RhoC-siRNA, CCN1-siRNA and CCN2-siRNA-transfected cells responding to Cd at 20 μmol/L for 6 h, determined by multiscan spectrometry (*n* = 6) and ICP-MS assay (*n* = 4), respectively. **f**, **g** HepG2 cells were pretreated with LY294002 for 1 h, and then the cellular Ca^2+^ influx **f** and Cd mass **g** after treatment of Cd at 20 μmol/L for 6 h was examined by multiscan spectrometry (*n* = 6) and ICP-MS assay (*n* = 4), respectively. **h**, **i** Scrambled control cells and MT1DP^low^ cells were transfected with RhoC, CCN1, CCN2, and AKT overexpression constructs for 24 h prior to Cd treatment at 20 μmol/L for 6 h, and thereafter cellular Ca^2+^ influx **h** and Cd mass **i** were determined by multiscan spectrometry (*n* = 6) and ICP-MS assay (*n* = 4), respectively

PI3K-AKT signaling crucially governs many downstream targets that are involved in proliferation, survival, homeostasis, and other important biological processes. We further endeavored to dig out the specific target(s) responsible for MT1DP-enhanced cell death upon the motivation by RhoC through CCN1/2-AKT pathway under Cd toxicity. It was inferred that Cd could be transported into cells through metal channels (in particular dependent on Ca^2+^ channel) in an ionic mimicry mechanism^6^. Meanwhile, the mass of cellular Cd accumulation fundamentally dictates the extent of its cytotoxicity^6, 37, 38^. Our results displayed that Ca^2+^ influx was elevated in scrambled control cells upon Cd treatment (*P* < 0.05); however, this elevation was markedly compromised by a Ca^2+^ channel antagonist verpamil (Fig. S12A, *P* < 0.05). As a consequence, Cd-induced cell death was significantly reversed by 70% upon the blockade of Ca^2+^ influx with verpamil pretreatment (Fig. S12B, *P* < 0.05), indicating that Cd-induced cytotoxicity was partially ascribed to increased Ca^2+^ influx. In addition, Rho GTPase family members could increase Ca^2+^ influx^39^ and CCN and AKT were also reported to activate l-type Ca^2+^ channel^40–42^. Based on these understandings, we thus hypothesized that MT1DP-conducted cellular signaling might modulate Cd-induced cell death through enhancing Ca^2+^ influx coupled to cellular Cd uptake. To address the hypothesis, Ca^2+^ influx and cellular Cd uptake were sternly assessed. As shown in Fig. 4a, Cd treatment-induced Ca^2+^ influx in scrambled control cells was markedly attenuated by MT1DP^low^ cells (*P* < 0.05). Analogously, the intercellular Cd mass was declined by about 30 and 20% in MT1DP^low^ cells at 6 and 24 h post Cd treatment, respectively, compared with that in scrambled control cells (Fig. 4b, *P* < 0.05). On the reverse, an approximately 35% increase of intercellular Cd mass was found in MT1DP^high^ cells at 6 h after Cd treatment, and, to a greater extent, a 50% increase was demonstrated in 24 h after Cd treatment, compared with that in vehicle control cells (Fig. 4c, *P* < 0.05). Similarly, reduced Ca^2+^ influx (15–20%) together with Cd mass (20–30%) were observed in cells with RhoC, CCN1, and CCN2 knockdown and in cells upon AKT inhibition (Figs. 4d–g, *P* < 0.05). To further confirm the role of RhoC, CCN1, CCN2, and AKT in regulating MT1DP-dependent Ca^2+^ influx and cellular uptake of Cd, RhoC, CCN1, CCN2, and AKT overexpression were carried out. As shown in Figs. 4h, i, exotic expression of RhoC, CCN1, CCN2, and AKT enhanced Ca^2+^ influx and intercellular Cd accumulation by approximate 10–45% in both scrambled control cells and MT1DP^low^ cells, compared with vehicle control cells (*P* < 0.05). Furthermore, RhoC, CCN1, CCN2, and AKT overexpression associated Ca^2+^ influx and cellular uptake of Cd were significantly undermined upon simultaneous MT1DP knockdown, namely in MT1DP^low^ cells relative to scrambled control cells (Figs. 4h, i, *P* < 0.05), pinpointing the important role of MT1DP in governing the RhoC-CCN1/2-AKT axis. These data together suggested that the cellular signaling activated by MT1DP in response to Cd further facilitated the cellular uptake of Cd coupled to accelerated Ca^2+^ influx.

### An inter-regulation between MT1H and MT1DP responding to Cd stress to promote cell death

**Fig. 5:**
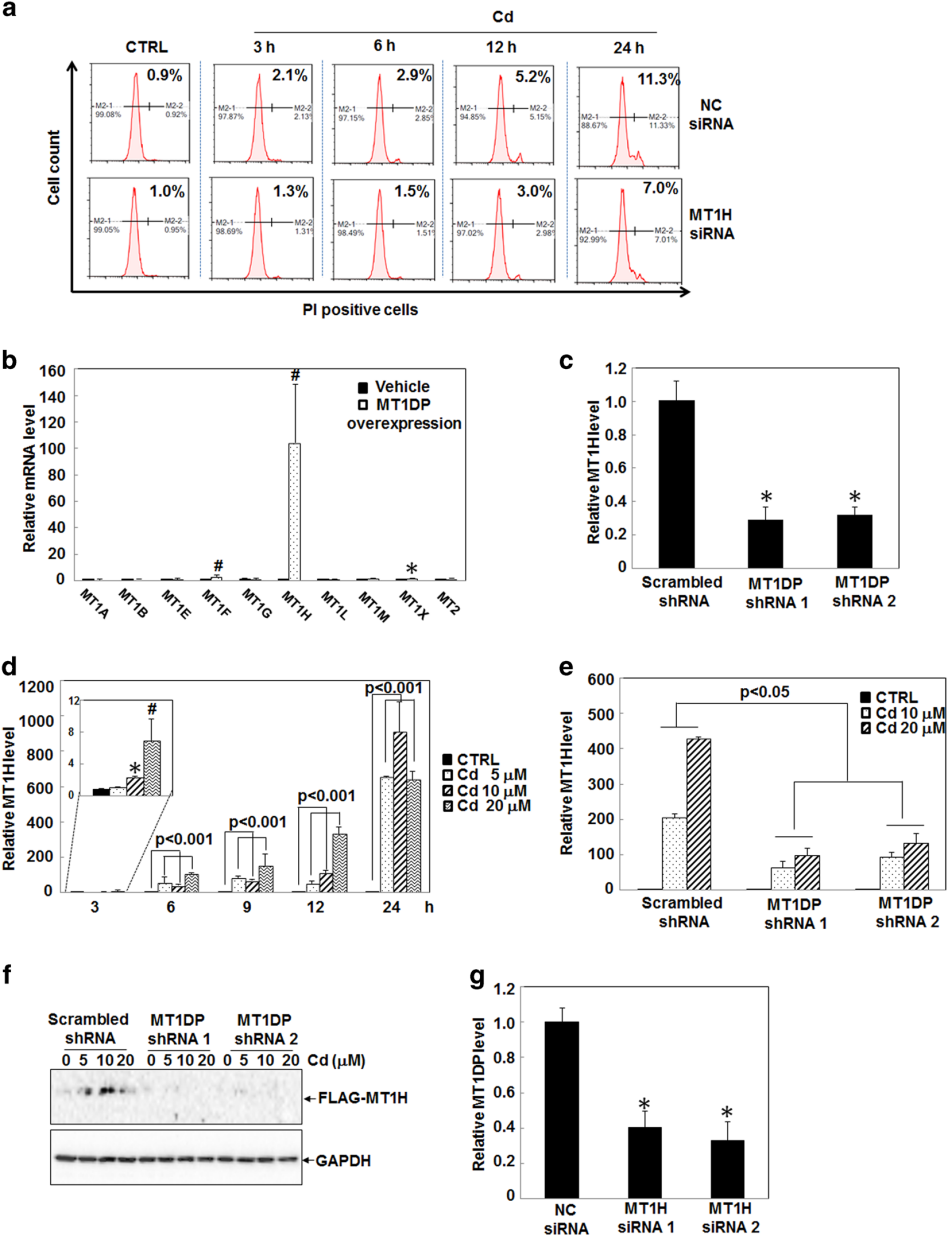
MT1DP regulates its parental gene MT1H level through a ceRNA mechanism. **a** Cell death analysis through flow cytometry analysis with PI staining in scrambled control and MT1H^low^ cells upon Cd treatment at 20 μmol/L for 3, 6, 12, and 24 h (*n* = 3). **b** qRT-PCR analysis of relative mRNA levels of MT1 family numbers in MT1DP overexpressed HepG2 cells (*n* = 3). **c** qRT-PCR assay of MT1H expression levels in scrambled control and MT1DP^low^ cells (*n* = 3). **d** MT1H mRNA levels in HepG2 cells in response to Cd over time, determined by qRT-PCR (*n* = 3). **e** qRT-PCR determination of MT1H mRNA levels in scrambled control and MT1DP^low^ cells upon Cd treatment at 10 and 20 μmol/L Cd for 24 h (*n* = 3). **f** Exotic FLAG-MT1H CDS + 3ʹ-UTR construct was transfected into scrambled control and MT1DP^low^ cells for 24 h, and cells were treated with Cd at indicated concentrations for 6 h, followed by western blot analysis of FLAG-MT1H. **g** Endogenous MT1H mRNA levels were knocked down by two sets of selective siRNA molecules in HepG2 cells, and then MT1DP levels were assayed by qRT-PCR analysis (*n* = 3)

Growing evidence suggests that pseudogenes and their cognate genes could act as ceRNAs to compete for complementary miRNAs in order to modulate each other’s mass^43, 44^. Given that most MT members, such as MT1A, are known to lead an important role in resisting the cytotoxicity from Cd and other stimuli^5, 45^. However, the biological functions of other MT1 family members including MT1H are elusive thus far. Recent studies reported a tumor-suppressor activity of MT1H in prostate cancer and hepatocellular carcinoma^46, 47^ indicative of a novel role of MT1H in regulating cell survival. Therefore, a possible role of MT1H was assumed in regulating cell death. First, endogenous MT1H was significantly diminished in HepG2 cells by small interfering RNA (siRNA)-mediated knockdown (Fig. S13, *P* < 0.001). Analogous to the pro-cell death function of MT1DP (Fig. 5a), there was significant difference in Cd-induced cell death between NC-siRNA and MT1H siRNA (*P* < 0.05), in a chronological way, in particular at 24 h Cd treatment (*P* < 0.05). These results therefore suggested a good biological correlation between MT1DP and MT1H, which encouraged us to postulate that there might exist a ceRNA mechanism between MT1DP and MT1H, especially under Cd toxicity. To answer this question, we closely interrogated a likely inter-regulation of between and MT1H and MT1DP. As shown in Fig. 5b, overexpression of MT1DP brought about >100-fold increase of MT1H, with little expression changes were observed for other MT1 members. Similarly, the expression level of MT1H was significantly downregulated by approximately 70% upon MT1DP decline in MT1DP^low^ cells, compared with that in scrambled control cells (Fig. 5c, *P* < 0.05). In support of the above findings, the MT1H level was upregulated by >50-fold upon exogenous MT1DP overexpression in L02 cells (Fig. S14), highlighting the discovery that the regulation of MT1H expression by MT1DP similarly occurred in normal liver cells. To exploit the probable implication of MT1H in Cd-induced cell death, its expression profile and function in modulating cell death were assessed. As shown in Fig. 5d, a tremendous induction of MT1H expression was found overall in a time- and dose-dependent manner (*P* < 0.05), in parallel to the changes of MT1DP (Fig. 1b). However, this induction of MT1H was compromised by approximate 55% and 70% in MT1DP^low^ cells treated with Cd at 10 and 20 μM, respectively (Fig. 5e, *P* < 0.05). Consistently, the alteration of MT1H at the protein level was verified in scrambled control cells and MT1DP^low^ cells in response to Cd exposure at 10 and 20 μM (Fig. 5f). Vice versa, MT1H knockdown by two sets of selective siRNAs was demonstrated to cause a significant reduction of MT1DP level as well (Fig. 5g and Fig. S13, *P* < 0.05).

### MT1H and MT1DP promote each other via miR-214

**Fig. 6:**
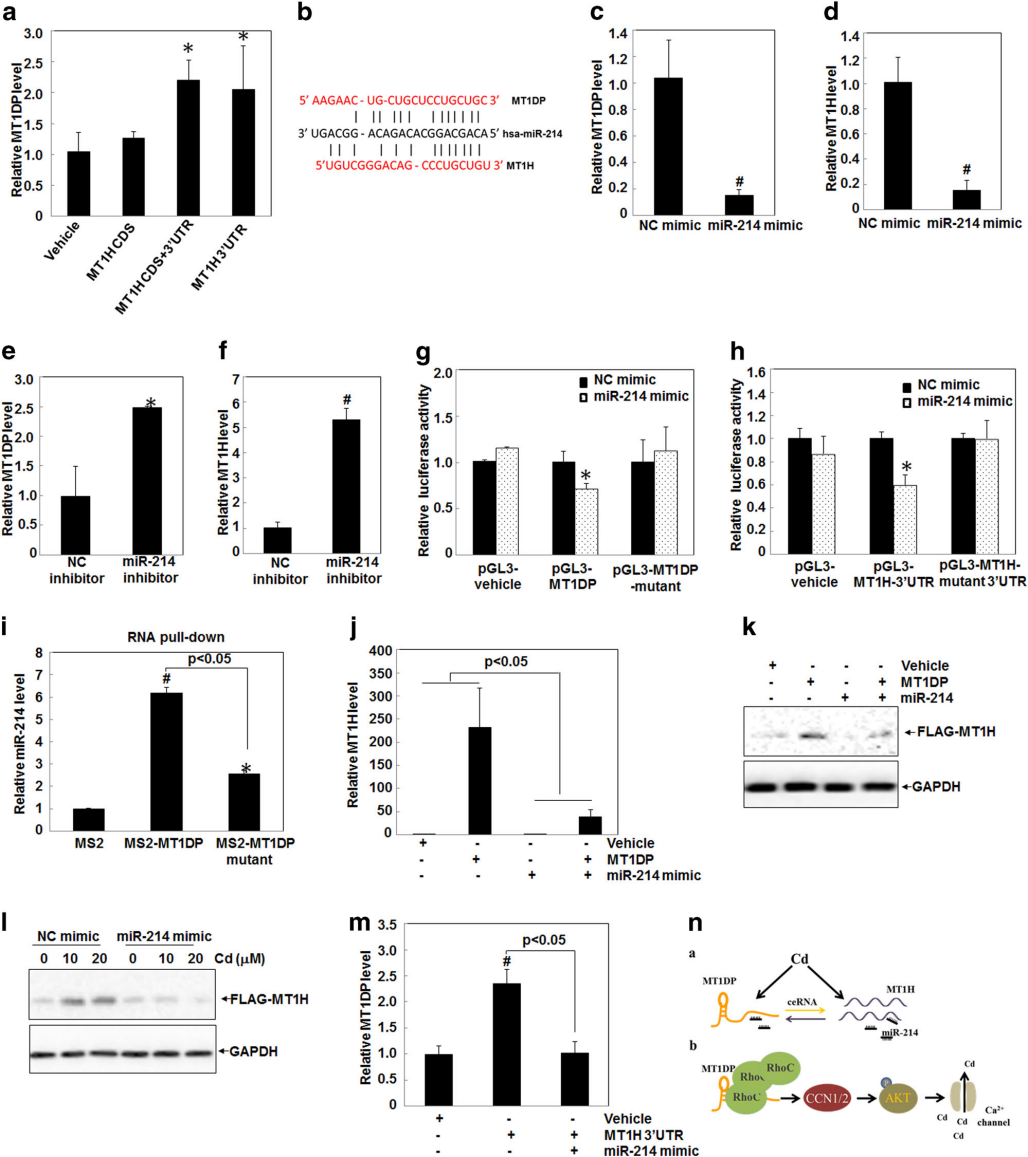
MT1DP competes for miR-214 with MT1H. **a** HepG2 cells were transfected with exotic FLAG-MT1H CDS, FLAG-MT1H CDS + 3ʹ-UTR, and FLAG-MT1H 3ʹ-UTR for 24 h, respectively, and then the MT1DP levels were measured by qRT-PCR (*n* = 3). **b** A schematic illustrating the putative target sites for MT1DP and MT1H in competing for miR-214. **c**–**f** Levels of MT1DP and MT1H were determined by qRT-PCR in cells transfected with NC-mimic and miR-214 mimic molecules **c**, **d** and NC-inhibitor and miR-214 inhibitor molecules **e**, **f** (*n* = 3), respectively. **g,** **h** Relative luciferase activities in HepG2 cells with expression of pGL3-vehicle, pGL3-MT1DP, and pGL3-MT1DP-mutant constructs **g** and pGL3-vehicle, pGL3-MT1H 3ʹ-UTR, and pGL3-MT1H 3ʹ-UTR-mutant constructs **h** upon transfection of NC-mimic and miR-214 mimic molecules, measured through the dual-luciferase assay (*n* = 3). **i** Relative enrichment of miR-214 in the pull-down lysates from cells using MS2-MT1DP and MS2-MT1DP mutant RNAs, respectively, examined by qRT-PCR assay (*n* = 3). Fold changes were normalized to U6 RNA levels. **j** The MT1H levels in HepG2 cells transfected with synthesized molecules of vehicle control, MT1DP, miR-214 and MT1DP + miR-214, detected by qRT-PCR assay (*n* = 3). **k** Western blot analysis of FLAG-MT1H in HepG2 cells. Cells were pre-transfected with FLAG-MT1H CDS + 3ʹ-UTR for 24 h, and were further transfected with synthesized molecules of vehicle control, MT1DP, miR-214 and MT1DP + miRNA-214 for another 24 h, followed by western blotting. **l** Similar to **k**, after pre-transfection of FLAG-MT1H CDS + 3ʹ-UTR for 24 h, changes of MT1H concentrations upon NC-mimic and miR-214 mimic molecules were determined by western blot analysis under Cd treatment at 20 μmol/L for 6 h. **m** qRT-PCR analysis of MT1DP levels in HepG2 cells transfected with MT1H 3ʹ-UTR and MT1H 3ʹ-UTR + miR-214 (*n* = 3). **n** A working model depicting the interplay of MT1DP with MT1H and RhoC to promote Cd-induced cellular toxicity

To elucidate the ceRNA mechanism between MT1H and MT1DP, we narrowed down the location of the binding site in MT1H mRNA by MT1DP. For this purpose, we constructed the vectors expressing MT1H-coding sequence (CDS) with or without its 3ʹ-untranslated region (UTR) sequence, respectively. As shown in Fig. 6a, MT1DP expression level was enhanced by approximately twofold in cells transfected with MT1H-CDS + 3ʹ-UTR and MT1H-3ʹ-UTR constructs, but not in cells transfected with MT1H-CDS construct (*P* > 0.05). This observation showed a positive regulation of MT1H 3ʹ-UTR in promoting MT1DP expression, and also further implied an inhibition on the blockade of MT1DP expression by according microRNAs (miRNAs). miRNAs take a primary role in the ceRNA mechanism by bridging lncRNAs for fine-tuned modulation for each other^48^ and previous studies also manifested that miRNAs link the pseudogenes with their ancestral genes to regulate the latter’s function^43, 44^. Therefore, we endeavored to excavate the linking miRNA(s) that would build up a bridge between MT1H and MT1DP.

For this aim, we predicted the putative miRNAs that are able to bind MT1DP and MT1H-3ʹ-UTR using online software miranda (http://www.microrna.org/microrna/home.do). As shown in Fig. 6b, there was only one same miRNA-binding site: miRNA-214, for both MT1DP and MT1H-3ʹ-UTR, displaying a possible function of miR-214 to bridge MT1H and MT1DP. To examine this hypothesis, the MT1DP and MT1H levels were assessed upon miR-214 mimics and inhibitors. On one hand, miR-214 mimics reduced the levels of both MT1DP and MT1H by ~80% (Figs. 6c, d, *P* < 0.001). On the other hand, miR-214 inhibitors enhanced the levels of MT1DP and MT1H by more than two- and fivefold (Figs. 6e, f, *P* < 0.05), respectively, compared with scrambled control cells. These observations signified the important role of miR-214 in regulating the stability of both MT1DP and MT1H.

To corroborate this finding, we examined the ability of miR-214 to maintain the stability of MT1DP and MT1H through luciferase assay. As shown in Fig. 6g, the luciferase activity of pGL3-MT1DP was significantly decreased by 28% in cells transfected with miR-214 mimics, relative to that in cells transfected with scrambled mimic molecules (*P* < 0.05). However, this decrease was abolished for the luciferase reporter with miR-214-binding site mutation in MT1DP (Fig. 6g and Fig. S15A, *P* > 0.05). Analogously, the luciferase activity of pGL3-MT1H-3ʹ-UTR greatly dropped by 40% in cells upon transfection of miR-214 mimics, compared with that in cells upon transfection of scrambled molecules (Fig. 6h, *P* < 0.05). Consistently, this drop was reversed for the reporter with mutation of miR-214-binding site in MT1H 3ʹ-UTR (Fig. 6h and Fig. S15B, *P* > 0.05). These results suggested the vital regulation of miR-214 on the stability of both MT1H and MT1DP. In other words, our results unearthed the role of MT1H in enhancing MT1DP stability by acting as a ceRNA to compete for the binding of miRNA-214.

To further substantiate the direct binding of miR-214 to MT1DP in cells, RNA pull-down was carried out in cells with overexpression of MS2 hairpin-tagged MT1DP. As shown in Fig. 6f, miR-214 was preferentially enriched in the MT1DP-MS2 pull-down complex, with about six times greater than that in NS-MS2 pull-down complex (*P* < 0.001). Meanwhile, this enrichment of miR-214 was drastically diminished by approximately 60% in MT1DP-MS2-mutant (MT1DP sequence with miR-214-binding site mutation) pull-down complex (Fig. 6i, *P* < 0.05). Nonetheless, MT1DP overexpression and knockdown elicited no effect on the cellular content of miR-214 (Fig. S16A and B, *P* > 0.05), excluding a direct regulation of MT1DP on miR-214 expression. Notably, the induction MT1H by MT1DP was greatly inhibited by miR-214 mimics at the mRNA and protein levels (Figs. 6j, k, *P* < 0.05). Consistent with this result, Cd-induced MT1H enhancement was also markedly compromised by miR-214 mimics (Fig. 6l). Furthermore, MT1H 3ʹ-UTR overexpression-induced MT1DP upregulation was also significantly attenuated by miR-214 mimics (Fig. 6m). Taken together, these data unearthed that miR-214 played an essential role in regulating the stability of both MT1H and MT1DP, and also revealed that MT1H and MT1DP in fact mutually shielded each other through acting as a reciprocal ceRNA to compete for miR-214.

### MT1H partially contributes to Cd-induced RhoC-CCN1/2-AKT pathway activation through miR-214 upon Cd

To further fill in the knowledge gap on whether MT1H also regulated RhoC-CCN1/2-AKT pathway, we looked into the changes of RhoC, CCN1, CCN2, and AKT phosphorylation in scrambled control cells, MT1DP^low^ cells and MT1H^low^ cells upon Cd treatment. As shown in Fig. S17, Cd-induced accumulation of protein mass for RhoC, CCN1, CCN2, and AKT activation were largely compromised in MT1DP^low^ cells, in contrast to a slight decline in MT1H^low^ cells. In addition, MT1DP overexpression also triggered greater activation of RhoC-CCN1/2-AKT signaling compared with MT1H CDS + 3ʹ-UTR construct (Fig. S18A), indicating that MT1DP in deed contributed much more to the activation of the RhoC signaling than MT1H. Moreover, we investigated the likely interaction between MT1H and RhoC. As shown in Fig. S19, an Ab against FLAG, but not IgG, could successfully pull-down MT1H in the cell lysate from cells transfected with FLAG-MT1H. However, RhoC protein was not visualized in FLAG-MT1H pull-downed cell lysate, ruling out a direct physical interaction of MT1H with RhoC. To further explore whether MT1H also regulates MT1DP-mediated activation of RhoC-CCN1/2-AKT pathway through miR-214, the only binding site within the 3ʹ-UTR of MT1H was mutated for regulation examination. Given that there is no miR-214-binding site within the CDS of MT1H, we therefore overexpressed miR-214 resistant form of MT1H CDS + 3ʹ-UTR to answer whether it may affect Cd-induced cell death and the activation of MT1DP/RhoC-CCN1/2-AKT signaling pathway. As shown in Fig. S18A, compared with the efficacy of MT1DP, MT1H CDS + 3ʹ-UTR overexpression only mildly increased the protein concentrations of RhoC, CCN1, and CCN2 and the phosphorylation level of AKT in cells responding to Cd treatment. Nevertheless, once the miR-214-binding site of MT1H CDS + 3ʹ-UTR was mutated, the RhoC-CCN1/2-AKT pathway activation was attenuated (Fig. S18A), indicating that MT1H necessarily but slightly contributed to the activation of RhoC-CCN1/2-AKT pathway upon Cd treatment. Additionally, MT1H CDS + 3ʹ-UTR overexpression significantly enhanced Cd-induced cell death by nearly twofold (*P* < 0.05), and this induction could be partially repressed when the miR-214-binding site was mutated (*P* < 0.05; Fig. S18B), stressing that MT1H was implicated in promoting Cd-induced cell death via inter-regulation between MT1DP and the intermediator miR-214.

## Discussion

Irrespective of acute and chronic exposure, liver is the primary site for Cd deposition, leading to inevitable liver injuries that account for Cd-associated morbidity and even lethality^7, 49^. MTs (mainly MT1 and MT2) are immediately synthesized by hepatocytes, and Cd-MT complex is also mainly formed in the cytosol of hepatocytes^5, 7, 50^. As a consequence of hepatocyte damage, Cd-MT complex is thereafter released into circulation and then delivered to other organs, such as kidney^49, 51^. In terms of Cd-induced hepatotoxicity, oxidative stress stemming from Cd ions and associated impairments to organelles and molecules (including DNA) are believed to be the main molecular basis^2, 52, 53^. Meanwhile, existing data indicate that Cd treatment also changed the epigenetic regulations that contributed to Cd toxicity, including altered DNA methylation and deregulation of microRNAs (e.g., miR-146a)^54, 55^. Compared with other epigenetic mechanisms, emerging evidence commences to recognize lncRNAs as important cellular machinery for stress responses to exotic harmful substances^11, 13^ although the current understanding is fairly obscure. In the current study, we uncovered an important role of a lncRNA, MT1DP, in potentiating Cd toxicity in hepatocytes.

Given that MT1/2 proteins play an essential role in detoxification under Cd-induced hepatotoxicity^5, 7^, whether some MT family members also contribute to aggravating cell death of hepatocytes has remained a mystery for years. In other words, whether the MT members overall govern an equilibrium between cellular defense and cytotoxicity upon Cd remains unknown. In the current study, a non-coding member in the MT family, MT1DP, was identified to calibrate the cellular machinery to shunt the cellular defense to cytotoxicity upon Cd stress through crosstalk with MT1H and RhoC. MT1DP is evolutionarily expressed in primates only including human and chimpanzee, but not present in the genome of rodents, such as mouse and rat^18, 20^. In other words, no equivalent analog of MT1DP is available in mouse and rat genomes, suggesting a vital role of MT1DP in orchestrating the cellular defense machinery in humans. Upon Cd stress, MT1DP was quickly and robustly induced and chronologically promoted cell death later on by enhancing Cd toxicity dependent on the activation of RhoC-CCN1/2-AKT signaling and consequently acceleration of Ca^2+^ influx. Moreover, another protein-coding member of the MT family, MT1H, was recognized to enhance the function of MT1DP through a ceRNA mechanism by competing with a common miRNA, miR-214. This study opens a new avenue to understand the biological functions of pseudogenes and elucidate their inter-regulation with protein-coding members in order for fine-tuning the cellular machinery.

Pseudogenes represent a subset of lncRNAs, which possess similar sequence with their parental genes but lack the ability to encode proteins^16, 56^. As a pseudogene, MT1DP used to be considered as a junk gene without biological functions^57^. Similar to regular lncRNAs as decoyer, guider or scaffold to interact with partners in order to alter their partners’ biological functions^9^ our results demonstrated that a small G protein, a member of GTPases, RhoC, was a binding partner of MT1DP, and the protein stability of RhoC was exclusively protected by MT1DP to prevent its lysosomal degradation, independent of ubiqutin–proteasome system, defining a role of MT1DP in regulating the protein turnover rate of RhoC. This is the first report on the regulation of RhoC protein stability through the lysosome-dependent system. Different from this mechanism, another Rho family number RhoA was found to be subject to degradation through ubiqutin–proteasome system and lysosomal system as well^58–60^. As a vital small GTPase, RhoC critically governs cell survival and differentiation by regulating a number of downstream effectors, such as Rhotekin (a scaffold protein that interacts with GTP-bound Rho proteins), IQ motif containing GTPase-activating protein 1 (IQGAP1; Ras GTPase-activating-like protein), and Rho-associated coiled-coil containing protein kinases (ROCKs; Rho-associated protein kinases)^32, 61, 62^. In the current study, CCN1 and CCN2 were uncovered to be the downstream targets of MT1DP/RhoC complex, and CCN1/2 was further enforced to activate AKT phosphorylation upon Cd treatment in hepatocytes. CCN1 and CCN2 are two members in the CCN family, and they have high sequence homology^63^ with similar biological functions including wound healing, inflammation and fibrogenesis, migration, embryonic development through regulating a variety of signaling pathways^63–65^. In accordance with our current findings, previous studies also demonstrated that CCN1 and CCN2 are early-response genes subject to the regulation by Rho GTPase through signaling pathways including integrin, p38, AP1, MRTF-A, and Smad4 in various cell types upon diverse stresses^63, 66, 67^. Furthermore, CCN1 and CCN2 were demonstrated to act onto PI3K-AKT signaling in modulating cell death and migration^35, 36, 63^ highlighting our finding of the regulation of MT1DP/RhoC-CCN1/2 signaling on AKT activation. Although the exact mechanisms responsible for cellular uptake of Cd have not been established yet, most studies support that Cd is uptaken through other metal channels, mainly Ca^2+^ channel^6, 68–70^. In parallel to our current data, as the final executor downstream of AKT activation by MT1DP/RhoC-CCN1/2 in response to Cd, Ca^2+^ channel was accelerated to uptake more Cd ions. On the reverse, inhibition of MT1DP/RhoC-CCN1/2-AKT signaling attenuated Ca^2+^ influx and cellular uptake of Cd as well. Together with previous findings that Ca^2+^ influx blockage was often associated with proliferation inhibition and cell apoptosis induction in various types of cells^71, 72^, our currents results further pinpointed the important role of Ca^2+^ channel in importing Cd and conducting Cd toxicity.

Unlike other protein-coding members responsible for Cd detoxification, the biological function of MT1H is still obscure. Recent studies suggested a tumor-suppressing function of MT1H^46, 47^ and, other than this, its protective role against Cd toxicity and other functions in stress-associated biological processes have not been reported yet. Here, we unearthed a new function of MT1H in elevating MT1DP-promoted cell death caused by Cd treatment. Mechanistically, MT1H was found to act as a ceRNA to compete for a common miRNA: miR-214, with MT1DP. As Cd treatment also boosted the level of MT1H, like a sponge, increased MT1H consequently adsorbed more miR-214 in order to elevate the level of MT1DP. In fact, our data demonstrated MT1H and MT1DP mutually protected each other through acting as a reciprocal ceRNA to compete for miR-214. Additionally, our data manifested that MT1H slightly affected the activation of RhoC-based signaling pathway and Cd-induced cell death through miR-214, suggesting a little contribution of MT1H to the activation of MT1DP/RhoC-CCN1/2-AKT pathway and resultant cell death outcome via miR-214. Although such a mutual ceRNA mechanism between protein-coding genes and their pseudogenes has not been extensively investigated, increasing evidence supports our finding on the reciprocal ceRNA mechanism between pseudogenes and their parental genes through competing for common miRNAs^43, 73^ such as cytochrome P450 family 2 subfamily A member 6 (CYP2A6) and its pseudogene CYP2A7 and phosphatase and tensin homolog deleted on chromosome ten (PTEN) and its pseudogene PTENP1^74, 75^.

### Conclusions

To summarize, we uncovered a crucial role of an early-response lncRNA MT1DP in chronologically enforcing cell death in hepatocytes under Cd stress. Mechanistically, our results unearthed the molecular basis underlying MT1DP-dependent signaling to enhance Cd toxicity: MT1DP interacted and stabilized RhoC protein to activate CCN1/2-AKT pathway and subsequently facilitate Ca^2+^ influx, resulting in accelerated cellular Cd uptake coupled to enlarged Cd toxicity (Fig. 6n). In addition, MT1H was found to quickly respond to Cd exposure along with MT1DP, and these two members were identified to shield each other through a mutual ceRNA mechanism in order to exacerbate Cd-induced cell death in a positive feedback loop (Fig. 6n). Together, we here unveiled a mystery whether a pseudogene within the MT family, MT1DP, has actual biological functions by focusing on its partners that harbor important roles in regulating Cd-induced cellular defense. We uncovered that MT1DP functions to switch the cellular defense to cytotoxicity through hooking up a crosstalk between its two partners, namely MT1H and RhoC, under Cd stress. This study would open an avenue to understand the biological roles of pseudogenes in normal physiology and in stress, and to depict the inter-regulation between pseudogenes and their parental genes in orchestrating important biological processes.

## Materials and methods

### Cell culture and transfections

Human liver hepatocellular cell line HepG2 and hepatic cells L02 were purchased from the Cell Resource Center of the Institute of Basic Medical Sciences (CAMS, China). Cells were cultured in Dulbecco’s modified Eagle’s medium (Hyclone, CA, USA) supplemented with 10% bovine calf serum (Hyclone), 100 IU/mL penicillin, and 100 mg/mL streptomycin (Hyclone) in a humidified incubator at 37 °C with 5% CO_2_. Cells were transfected using Lipofectamine 2000 reagent according to the manufacturer’s instructions (Invitrogen).

### Plasmids and reagents

The human MT1DP-shRNA sequences were cloned into a lentiviral vector PLKO.1 according to the manufacturer’s instructions (Addgene) to construct MT1DP shRNA1 and MT1DP shRNA2 transfectants. WT MT1DP, WT MT1DP with mutation in binding site for miR-214 (substitute ATACA for CTGCT), as well as WT MT1H 3ʹ-UTR and MT1H 3ʹ-UTR with mutant sequences in binding site for miR-214 (substitute ATACA for CTGCT) were synthesized and accordingly cloned into the luciferase reporter vector PGL3-promoter to construct corresponding luciferase reporter transfectants. The MT1H CDS, MT1H 3ʹ-UTR and MT1D CDS + 3ʹ-UTR sequences were amplified from human complementary DNA (cDNA) and then cloned into pFLAG-CMV-2 expression vector to construct corresponding overexpression plasmids. The MT1DP cDNA sequence was cloned into pGEM-T and pCDNA3.0–12xMS2bs to obtain MT1DP overexpression and MT1DP-MS2 constructs, respectively. All primer sequences are shown in Supplementary Table 1. The pAd-RhoC-V14 expression plasmid was kindly provided by Professor Yan Wu at the School of Medical Science and Laboratory Medicine, Jiangsu University, Zhenjiang, Jiangsu, China. The pCDNA-12xMS2bs, and FLAG-2xMCP were kindly provided by Professor Xiaofei Zheng at Beijing Institute of Radiation Medicine, Beijing, China. The CCN1, CCN2, and AKT expression plasmids were purchased from Vigene Biosciences (Jinan, China). The NC miRNA mimic and miR-214 mimic and inhibitor RNA oligos, plus siRNA molecules for RhoC, CCN1, CCN2, and MT1H were purchased from Gene Pharma Bio-Technology (Shanghai, China). The primary antibodies (Abs) against RhoC, CCN1, CCN2, and p53 were purchased from Proteintech Group (Wuhan, China). The Abs against p-AKT, AKT, GAPDH, and HuR were purchased from Cell Signaling Technology (Beverly, MA, USA). Anti-FLAG Ab, MG132, CHX, BFA, verpamil and LY294002 were purchased from Sigma (St. Louis, MO, USA). DIG-11-dUTP was purchased from Roche (Basel, Switzerland).

### Inductively coupled plasma MS (ICP-MS) analysis

After treatment, HepG2 cells were harvest and washed with phosphate-buffered saline (PBS), followed by digestion with mixed acid using a microwave on a MARS machine (CEM Corp., Mattews, NC) for 24 h. The amounts of Cd in the collected cells were determined by ICP-MS using a quadrupole ICP mass spectrometer (Agilent, Tokyo, Japan), as previously described^76–78^.

### Cell death analysis by flow cytometry

After treatment, cell death of HepG2 cells were collected and washed with PBS, and collected were then subject to propidium iodide (PI) staining for 30 min. Finally, PI-positive cells were determined through flow cytometry analysis, as described previously^78, 79^.

### Luciferase activity assay

Post treatment, transfected cells were harvest and lysed with 1 × passive lysis buffer for 30 min at 4 °C. The luciferase activities were then measured using the Dual-Luciferase Reporter Assay Kit (Promega, Madison, MI) according to the manufacturer’s instructions.

### Quantitative reverse transcriptase–PCR (qRT-PCR) analysis

Total RNAs were extracted from cells with Trizol reagent (Invitrogen, USA), and then 2–5 μg RNAs were reverse-transcribed into cDNA with M-MLV reverse transcriptase. qPCR was performed on an iQ5 qRT-PCR instrument (Bio-Rad), as described previously^78, 79^ GAPDH was used as a loading control for normalization. The primer sequences are listed in Supplementary Table 1.

### RNA-sequencing analysis

Scrambled control cells and MT1DP^low^ cells were treated with or without Cd at 20 μM for 24 h, and then total RNAs were extracted. RNA-sequencing (RNA-seq) analysis was performed at The Beijing Genomics Institute (BGI) using Illumina HiSeq 2000 platform with paired-end 100-bp runs, as previously described^80^.

### Western blot analysis

Harvested cells were lysed, and total proteins were extracted with RIPA lysis buffer (Solarbio Inc., Beijing, China) containing protease inhibitor cocktail (Roche, Switzerland) for 30 min. Afterward, equal amounts of cell lysates were subjected to sodium dodecyl sulfate-polyacrylamide gel electrophoresis (SDS-PAGE), followed by blotting analysis, as described previously^78, 79^.

### RIP assay

RIP assay was performed using a Magna RIP™ RNA-binding protein immunoprecipitation kit (Millipore, Bedford, MA, USA) according to the manufacturer’s instructions. Briefly, cells were harvested after treatment, and collected cells were then lysed with RIP lysis buffer. Thereafter, 5 μg anti-RhoC Ab and normal rabbit IgG (Millipore) were incubated with Magnetic Beads Protein A/G for 1 h to form beads–Ab complexes that were used to precipitate RNAs in cell lysates. Co-precipitated RNAs were purified with phenol–chloroform–isoamyl alcohol (25:24:1) extraction after proteinase K digestion, and finally the target RNAs were detected by qRT-PCR.

### RNA-protein pull-down assay and protein identification

Linearized pGEMT-MT1DP was used as the template for MT1DP transcription, and MT1DP RNAs were then produced through in vitro transcription using Ambion mMESSAGE mMACHINE T7 Transcription Kit following the manufacturer’s instructions (Invitrogen). MT1DP RNAs were labeled with biotin, and proteins were pulled down from cell lysates using a RNA-Protein Pull-Down kit on the basis of manufacturer’s instructions (Thermo Fisher Scientific, USA). RNA-pulled down proteins were separated by SDS-PAGE, and then differentially presented bands were analyzed by MS at Beijing Protein Institute.

### FISH assay

Cells were washed with cold PBS three times for 10 min, and then fixed in 4% formaldehyde at room temperature for 10 min, followed by permeabilization in 0.5% Triton-100 at 4 °C for 10 min. Thereafter, cells were washed with PBS for three times prior to prehybridization with prehybridization buffer (Ribobio, Guangzhou, China) at 37 °C for 30 min. Afterward, cells were incubated with synthesized digoxygenin-11-dUTP (DIG)-labelled MT1DP FISH probes at 37 °C in the dark overnight in a humid chamber. Cells were then washed with 0.1% Tween-20/4×SSC for three times at 42 °C for 5 min each time, followed by 2×SSC and 1×SSC washing for 5 min for each at 42 °C. Then, cells were incubated with a FITC-anti-digoxin Ab (Jackson, PA, USA) for 1 h, followed by three washes with PBS, and were finally stained with 4ʹ,6-diamidino-2-phenylindole, dihydrochloride (DAPI) for 10 min at room temperature. Nuclei were counter-stained DAPI. Immunofluorescence was imaged on a confocal fluorescence microscope (Olympus, Japan).

### Ca^2+^ influx measurement

Ca^2+^-sensitive fluorescent dye Fluo-3AM was used for the measurement of cellular free Ca^2+^ content. In brief, after Cd treatment, HepG2 cells were incubated with 5 µM Fluo-3AM (Beyotime, Beijing, China) for 30± min at 37 °C, and then the fluorescence intensity of Fluo-3 AM probe was measured on a multiscan spectrometry using excitation 506 nm and emission 525 nm (Thermo Fisher Scientific, USA).

### RhoC activity assay

Cell lysates were incubated with 50 μl Rhotekin-RBD Agarose beads for 1 h at 4 °C, and then RhoC activity was assayed using a RhoC activation assay kit according to manufacturer’s instructions (Abcam). Precipitated GTP-Rho was detected by western blot analysis using an anti-RhoC Ab.

### Statistical analysis

All data are shown as mean ± standard deviation (SD), and statistical analysis was carried out with either independent *t*-test or one-way analysis of variance test. Experimental data were analyzed using the SPSS software. *P*-value less than 0.05 (*P < 0.05) or 0.001 (^#^*P* < 0.001) indicated statistically significant difference.

## Electronic supplementary material

MT1DP SUPPLEMENTAL MATERIAL R2
