# Supplementary material for: Long non-coding RNA MT1DP shunts the cellular defense to cytotoxicity through crosstalk with MT1H and RhoC in cadmium stress
Source: Cell Discov. 2018 Jan 30;4:5. doi: 10.1038/s41421-017-0005-y (PMC5824791; doi:10.1038/s41421-017-0005-y)
Supplement: Supplementary file 1 — MT1DP SUPPLEMENTAL MATERIAL R2 [file 41421_2017_5_MOESM1_ESM.docx]

**Supplementary Figures and Figure Legend.**

**
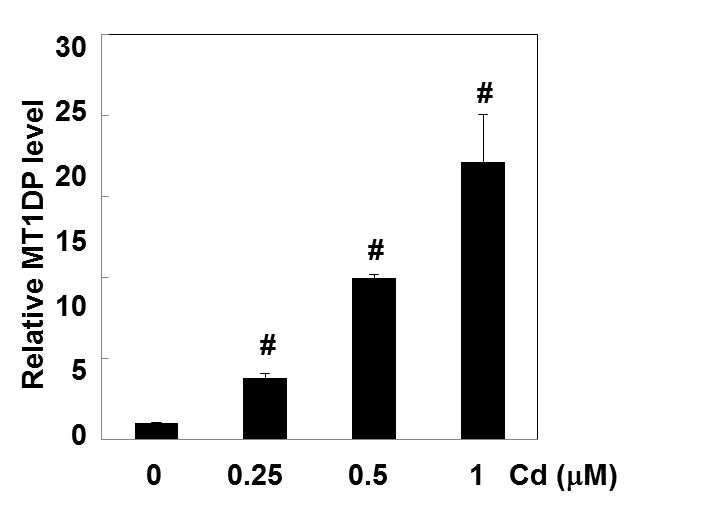
**

**Supplementary Figure 1. MT1DP is induced under low concentrations of Cd exposure.** HepG2 cells were treated with Cd at indicated concentrations for 24 h, and then the MT1DP levels were detected by qRT-PCR assay (n=3).

**
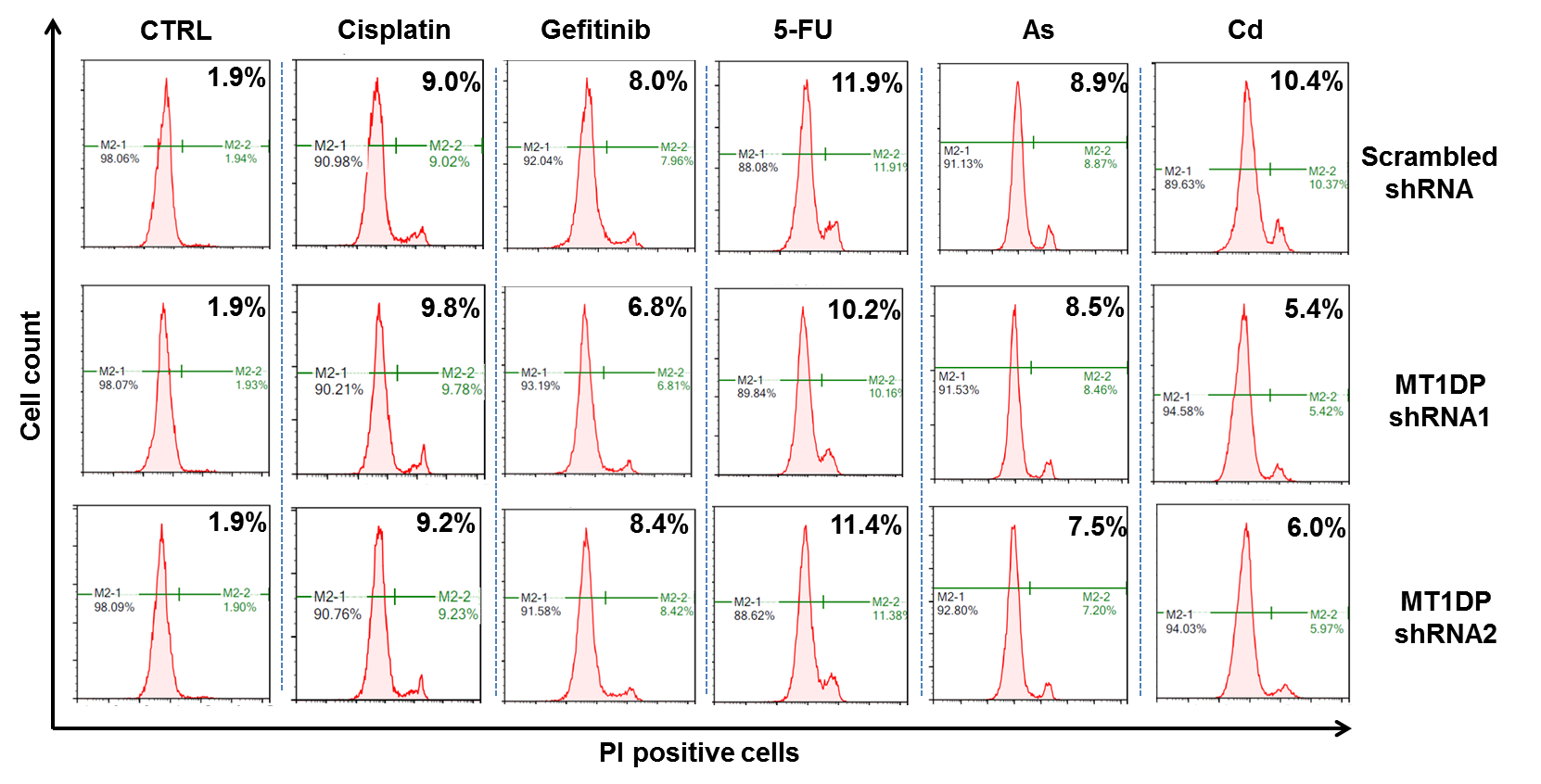
**

**Supplementary Figure 2. MT1DP contributes to Cd-induced cell death.** Cell death were compared through flow cytometry analysis with PI staining in scrambled control and MT1DP^low^ cells treated with 2 μg/mL cisplatin, 5 μM/mL gefitinib, 50 μg/mL 5-FU, 10 μmol/L As and 20 μmol/L Cd (n=3).


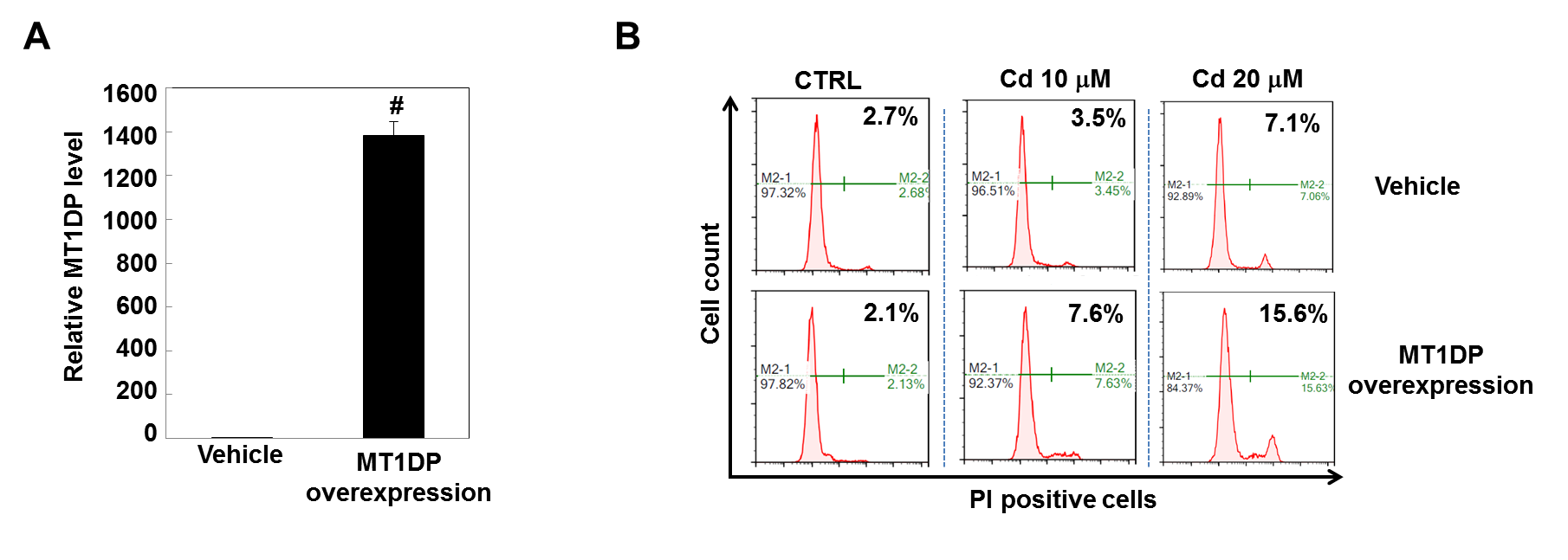


**Supplementary Figure 3. Cell death in MT1DP^high^ cells upon Cd.** (A) The level of MT1DP in vehicle control and MT1DP overexpression cells, detected through qRT-PCR assay (n=3). (B) The proportions of PI-positive cells for vehicle control and MT1DP overexpression cells in response to Cd for 24 h, assessed by flow cytometry analysis (n=3).


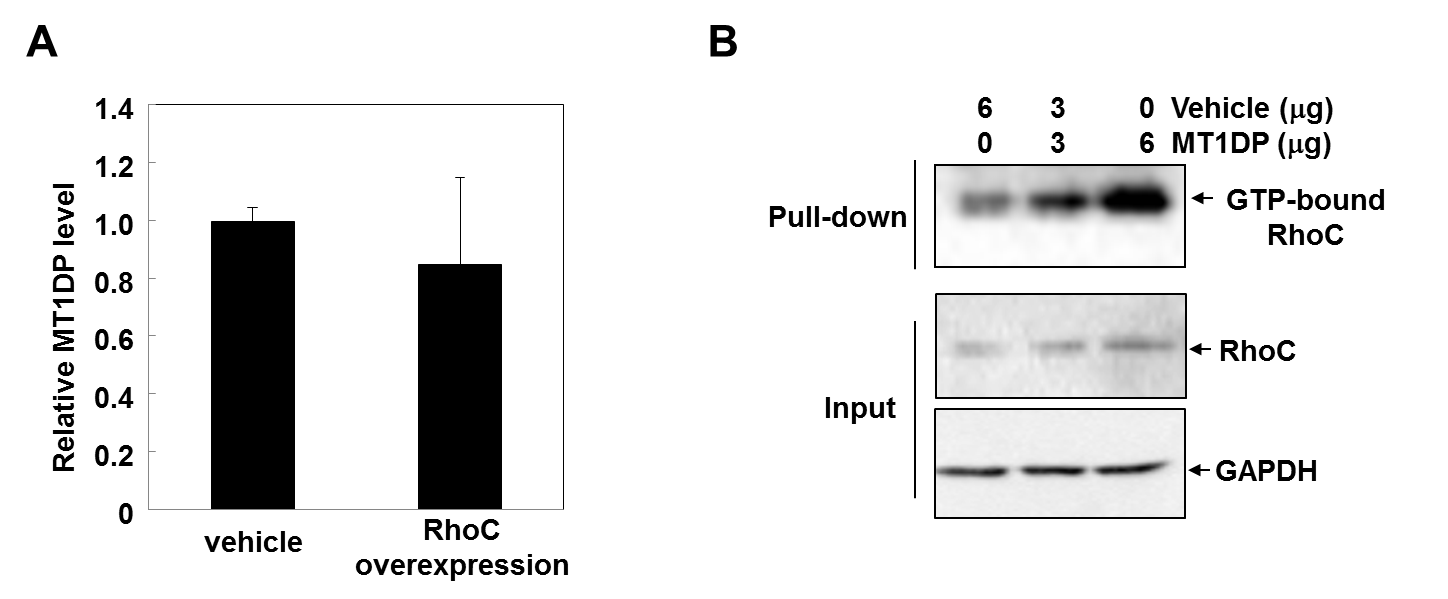


**Supplementary Figure 4. MT1DP regulates the activity of RhoC.** (A) Expression levels of MT1DP were compared by qRT-PCR assay in vehicle control and RhoC overexpression cells (n=3). (B) HepG2 cells were transfected with MT1DP overexpression plasmids at indicated concentrations for 48 h, and then GTP-bound RhoC was pulled down using Rhotekin-RBD agarose beads, followed by Western blot analysis.


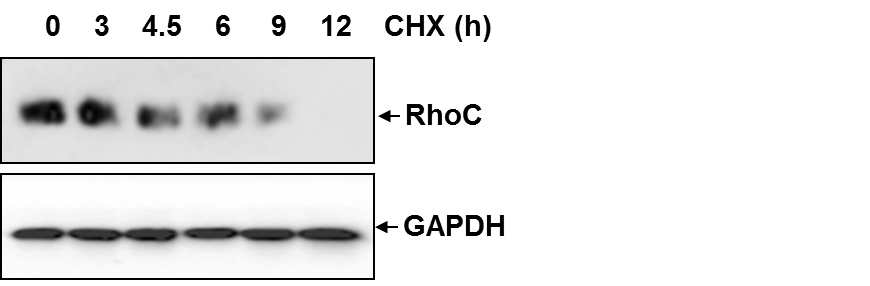


**Supplementary Figure 5.** **RhoC protein is unstable.** HepG2 cells were treated with 40 μmol/L CHX for indicated time, and then the mass of RhoC protein was characterized by Western blot analysis.


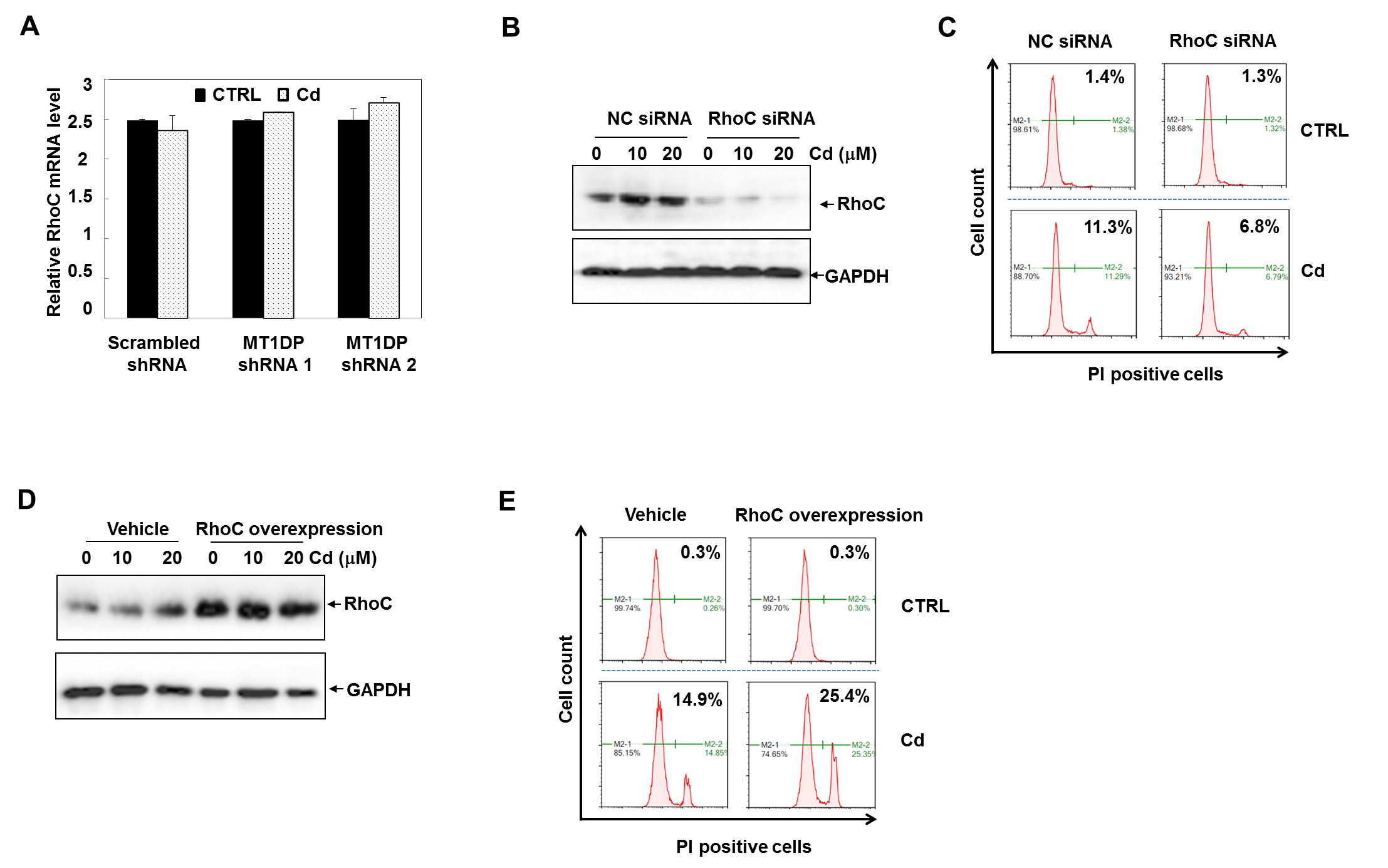


**Supplementary Figure 6. MT1DP governs the protein stability of RhoC to promote Cd-induced cytotoxicity.** (A) The mRNA levels of RhoC in scrambled-shRNA control cells and MT1DP^low^ cells in response to Cd at 20 μmol/L for 24 h, determined by qRT-PCR assay. (B) Protein concentrations of RhoC in scrambled-shRNA control cells and MT1DP^low^ cells under the treatment of Cd for 24 h, measured by Western blotting. (C) Flow cytometry analysis of cell death in Cd-treated NC siRNA- and RhoC siRNA- transfected cells with or without Cd treatment at 20 μmol/L (n=3). (D) Western blot analysis of the protein contents of RhoC in vehicle control and RhoC overexpressed cells upon Cd at indicated concentrations for 6 h. (E) The proportions of cell death in vehicle control and RhoC overexpression cells under Cd treatment for 24 h, determined by flow cytometry analysis with PI staining (n=3).

**
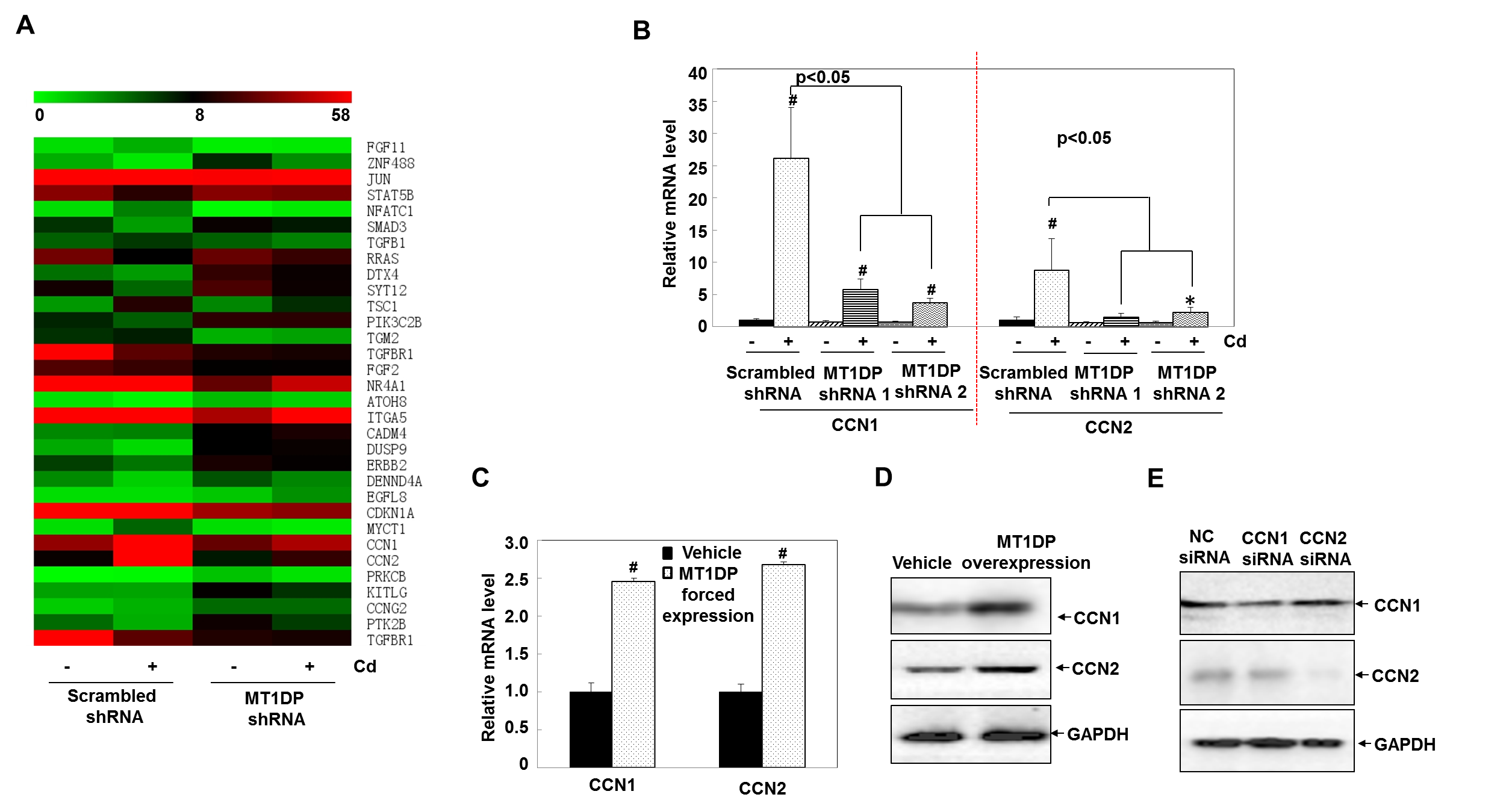
**

**Supplementary Figure 7. CCN1 and CCN2 are downstream targets of MT1DP/RhoC complex.** (A) Heatmap illustration of relative expression levels of 32 cell death-relevant genes based on RNA-Seq data in MT1DP^low^ cells relative to scrambled-shRNA control cells upon Cd treatment at 20 μmol/L for 24 h. (B) qRT-PCR analysis of relative mRNA levels for CCN1 and CCN2 in scrambled-shRNA control cells and MT1DP^low^ HepG2 cells in response to Cd at 20 μmol/L for 24 h (n=3). (C-D) The mRNA (C) and protein (D) levels of CCN1 and CCN2 in vehicle control and MT1DP overexpressed cells, determined by qRT-PCR (n=3) and Western blotting, respectively. (E) Western blot analysis of CCN1 and CCN2 in HepG2 cells transfected with according selective siRNA molecules.


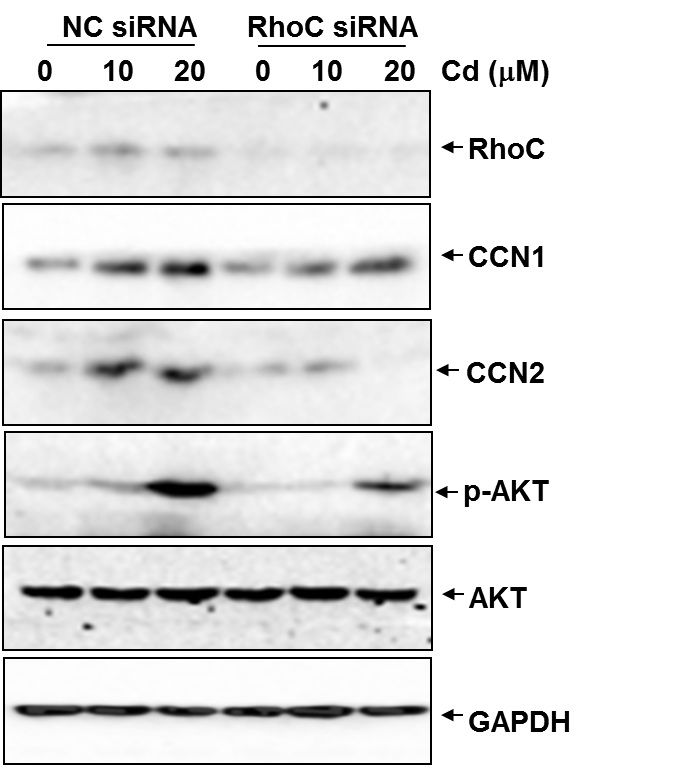


**Supplementary Figure 8. Activation of CCN1/CCN2-AKT pathway is regulated by RhoC.** RhoC, CCN1, CCN2 protein levels and AKT phosphorylation were measured by Western blot analysis in NC-siRNA and RhoC-siRNA transfected cells under Cd treatment for 24 h.


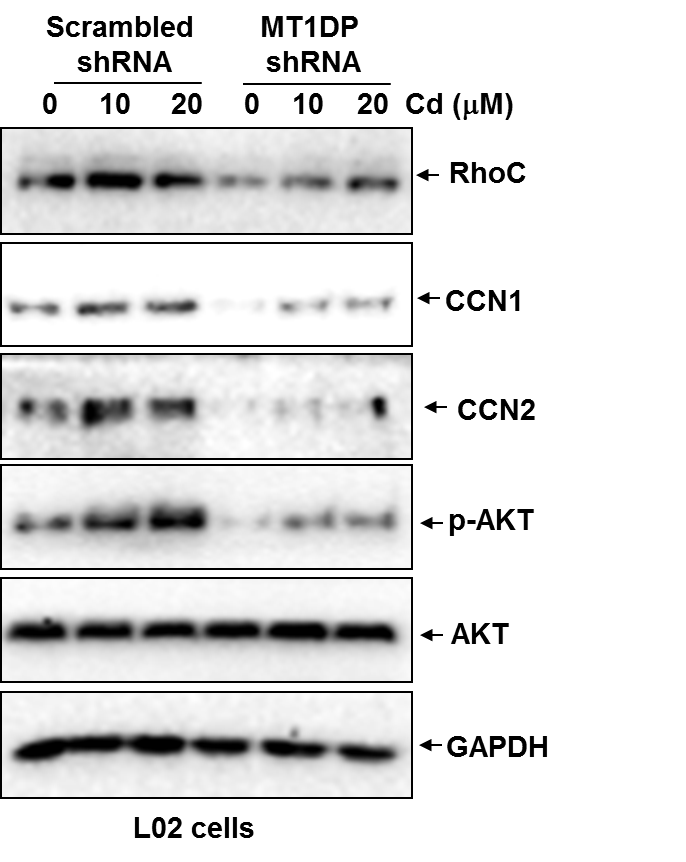


**Supplementary Figure 9. RhoC/CCN1-CCN2/AKT pathway is regulated by MT1DP in L02 cells**. The protein contents of RhoC, CCN1, CCN2 and phosphorylated AKT were measured by Western blot analysis in scrambled control and MT1DP^low^ L02 cells.


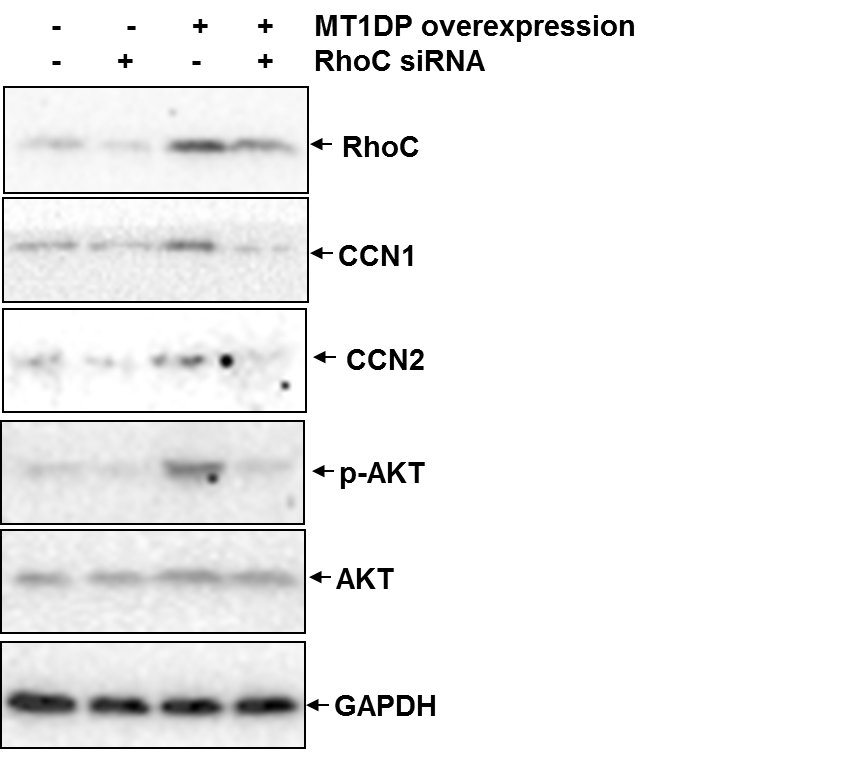


**Supplementary Figure 10. RhoC mediates the effect of MT1DP on the activation of CCN1-CCN2/AKT pathway.** HepG2 cells were transfected with RhoC siRNA molecules for 24 h, and were then transfected with MT1DP overexpression constructs for another 48 h. Afterwards, the protein contents of RhoC, CCN1, CCN2, phosphorylated AKT and total AKT were determined by Western blotting.


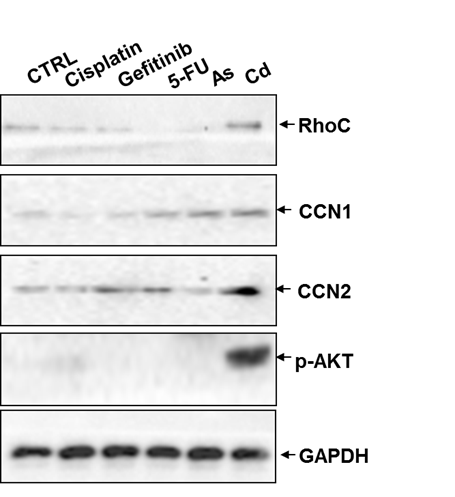


**Supplementary Figure 11. RhoC-CCN1/2-AKT pathway is selectively induced by Cd.** HepG2 cells were treated with 2 μg/mL cisplatin, 5 μM/mL gefitinib, 50 μg/mL 5-FU, 10 μmol/L As and 20 μmol/L Cd for 6 h, and then the protein concentrations of RhoC, CCN1, CCN1 and phosphorylated AKT were determined by Western blot analysis.


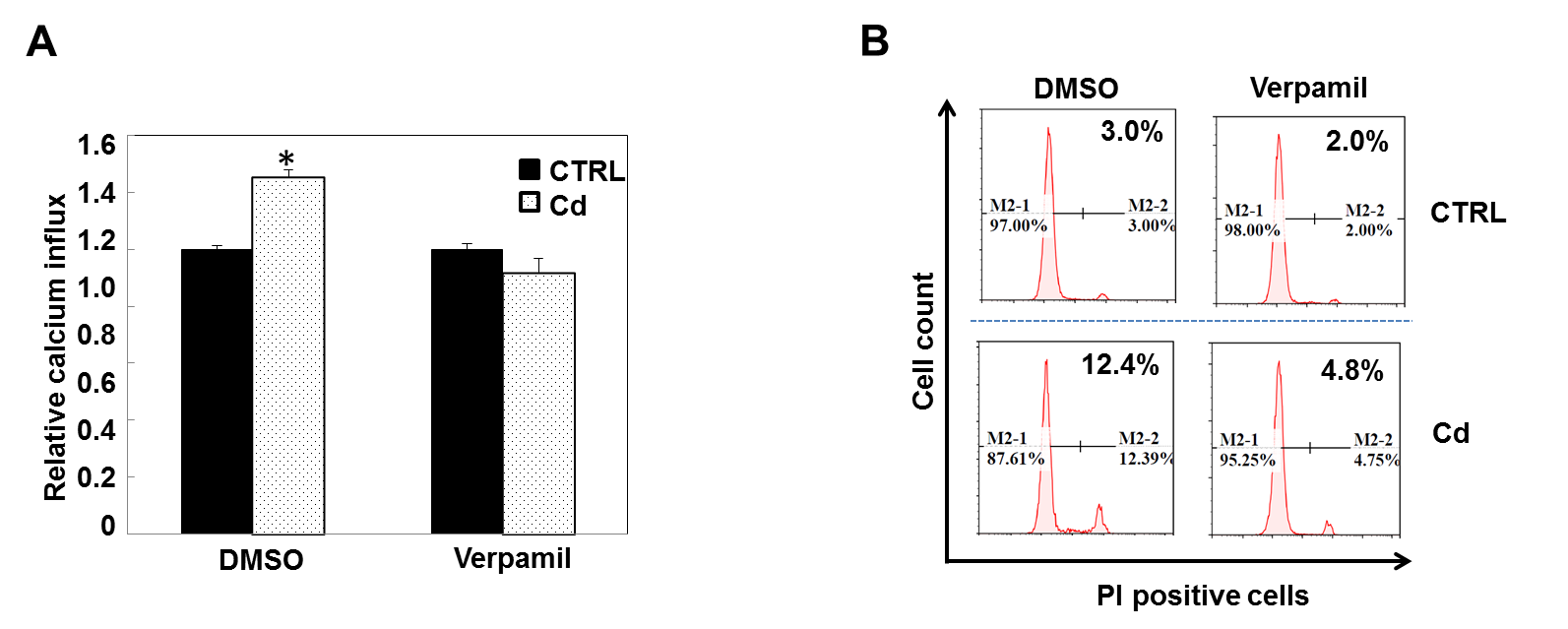


**Supplementary Figure 12. Ca^2+^ influx is indispensable for Cd-induced cell death.** (A) HepG2 cells were first pretreated with 10 μmol/L verpamil for 1 h prior to Cd treatment for 6 h, and cellular Ca^2+^ influx was then determined by multiscan spectrometry (n=6). (B) HepG2 cells were pretreated with verpamil at 10 μmol/L for 1 h prior to Cd treatment for another 24 h, and thereafter the proportions of PI-positive cells were determined by flow cytometry analysis (n=3).


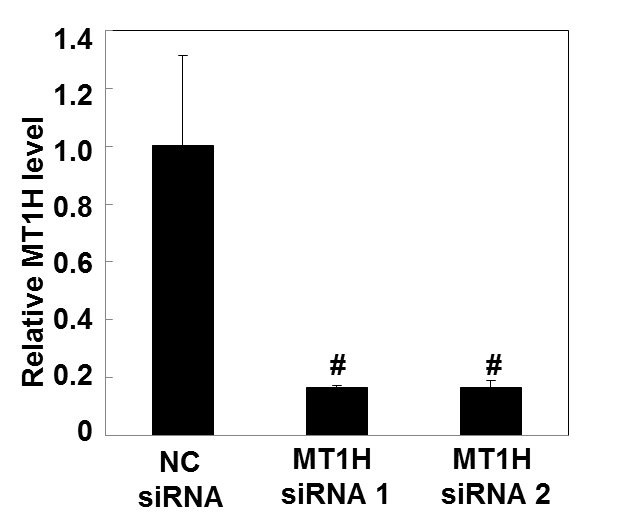


**Supplementary Figure 13. Efficiency of MT1H siRNAs.** mRNA level of MT1H in NC-siRNA control cells and MT1H-siRNA transfected HepG2 cells were compared by qRT-PCR assay (n=3).


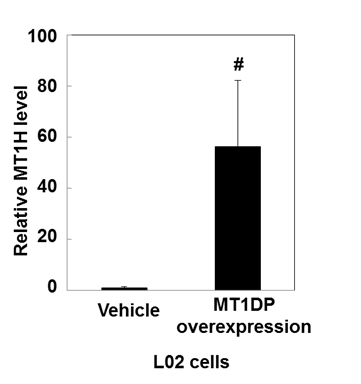


**Supplementary Figure 14. MT1DP regulates MT1H expression in L02 cells.** The mRNA levels of MT1H in vehicle- and MT1DP overexpression construct-transfected L02 cells were analyzed by qRT-PCR assay (n=3).

**
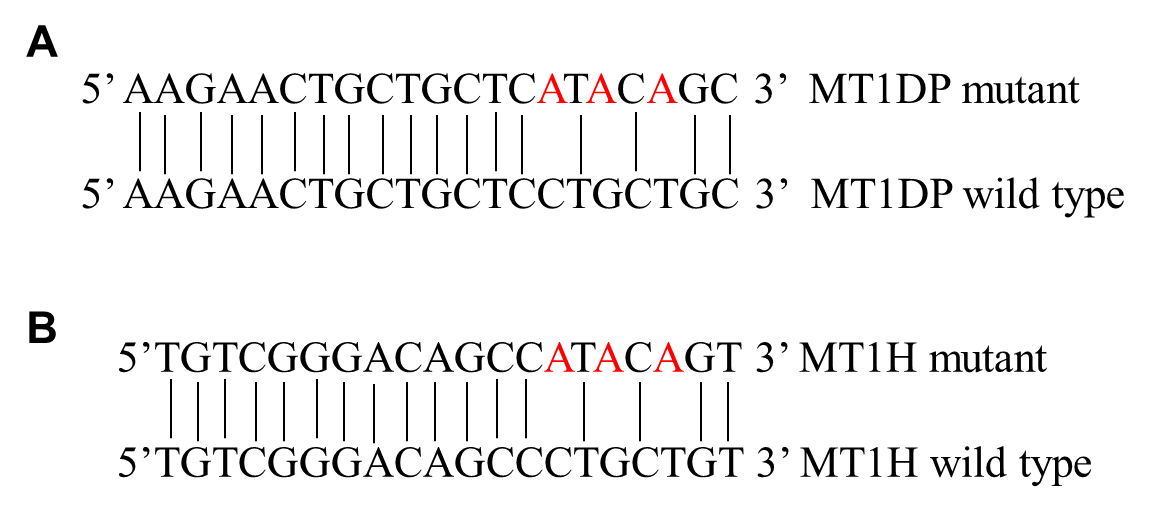
**

**Supplementary Figure 15. Illustration of mutant nucleotides within MT1DP and MT1H mRNAs.** Schematic delineating the mutant nucleotides in the sequences of pGL3- MT1DP (A) and pGL3-MT1H-3’UTR (B) plasmids.

**
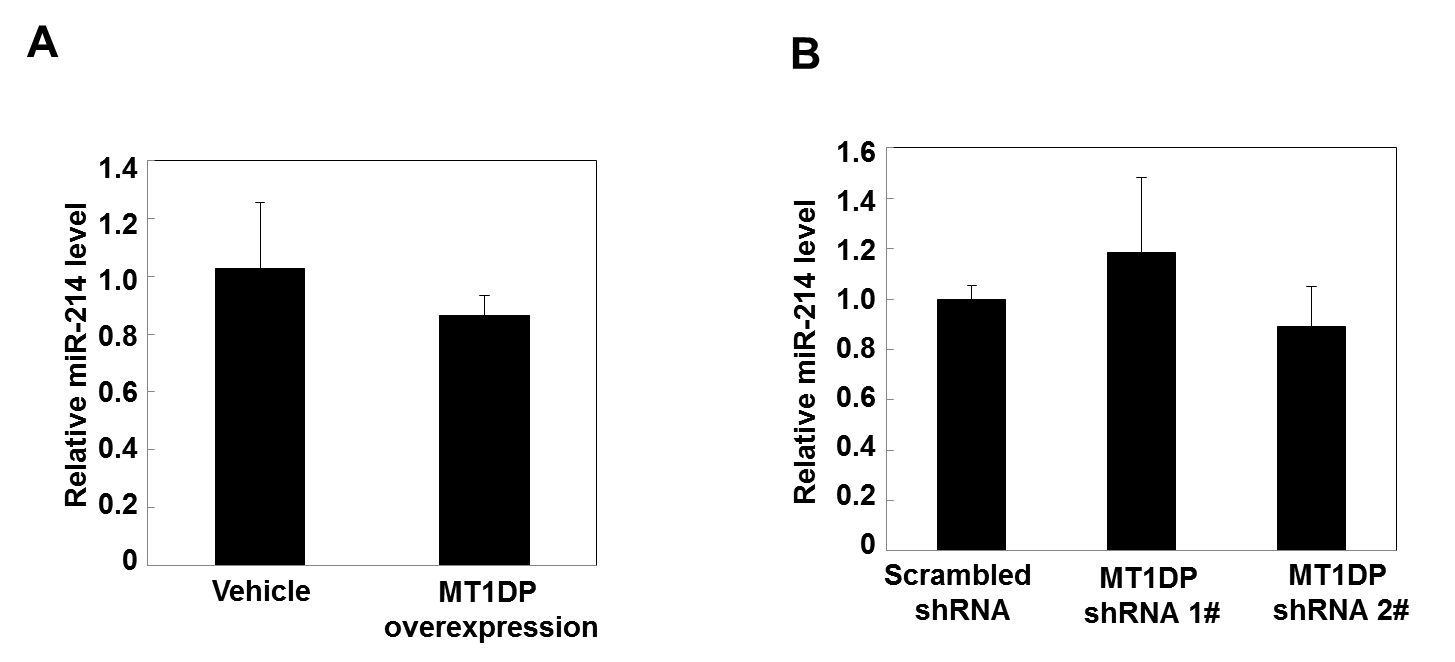
**

**Supplementary Figure 16. MT1DP elicited no effect on miR-214 content.** qRT-PCR assay of the levels of miR-214 in vehicle control and MT1DP overexpressed cells (A), and scrambled-shRNA control cells and MT1DP^low^ cells (B) (n=3).

**
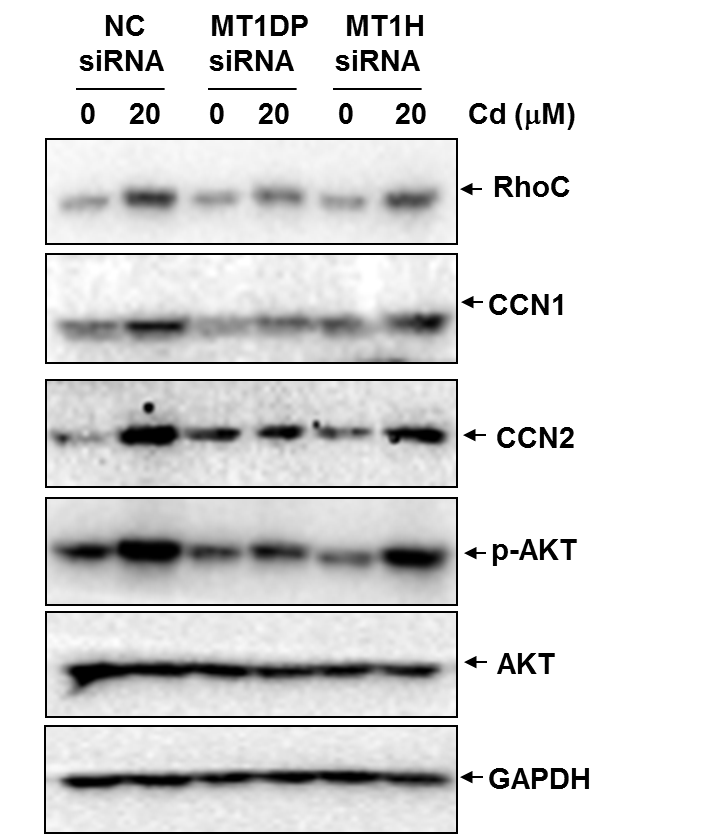
**

**Supplementary Figure 17. MT1DP and MT1H differentially modulate Cd-induced RhoC/CCN1-CCN2/AKT signaling.** Protein concentrations of RhoC, CCN1, CCN2 and AKT phosphorylation in scrambled-shRNA control cells, MT1DP^low^ cells and MT1H^low^ cells under the treatment of Cd for 6 h, as measured by Western blotting.


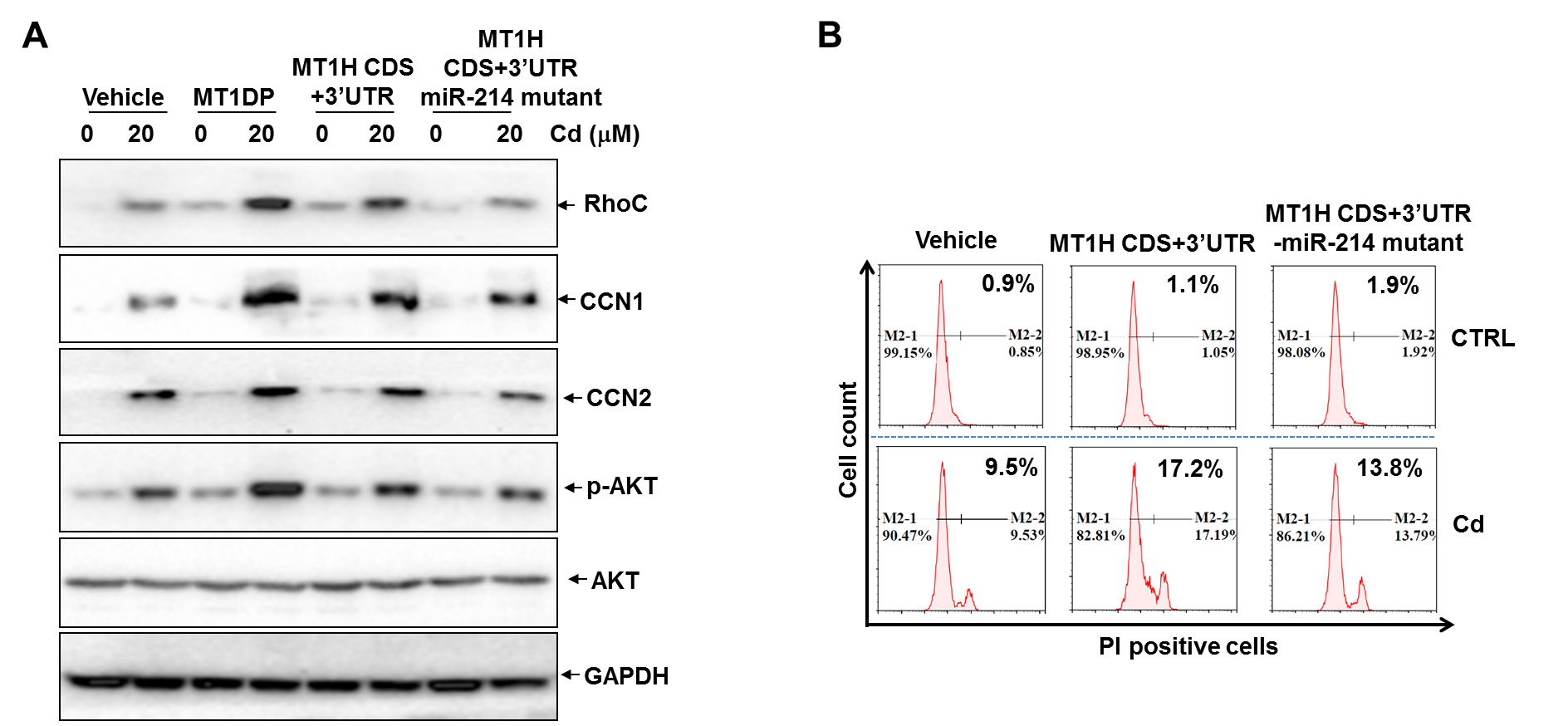


**Supplementary Figure 18. MT1H contributes to Cd-induced RhoC/CCN1-CCN2/AKT pathway activation and cell death upon Cd through miR-214.** (A) HepG2 cells were transfected with exotic MT1DP, MT1H CDS+3’UTR and MT1H CDS+3’UTR-miR214 mutant constructs for 24 h, followed by treatment of 20 μmol/L Cd for 6 h. Afterwards, the protein concentrations of RhoC, CCN1, CCN2 and phosphorylated AKT were analyzed by Western blot analysis. (B) HepG2 cells were transfected with exotic MT1H CDS+3’UTR and MT1H CDS+3’UTR-miR214 mutant constructs for 24 h, and cell death thereafter was assessed by flow cytometry analysis after Cd exposure at 20 μmol/L for 24 h.


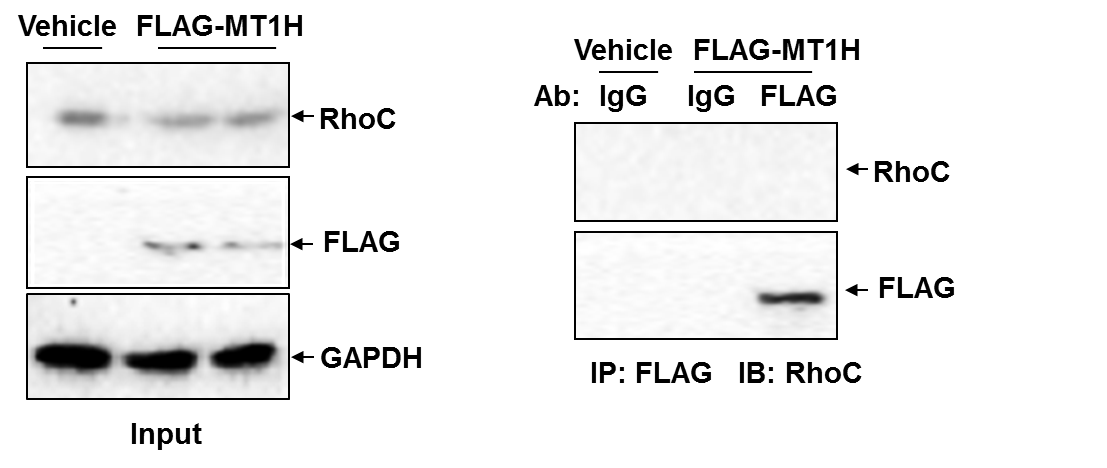


**Supplementary Figure 19. Interaction assessment between MT1H and RhoC.** HepG2 cells were transfected with vehicle and FLAG-MT1H constructs for 24 h, and thereafter an Ab against FLAG and normal IgG and were used for Co-Immunoprecipitation assay. Protein contents of RhoC and FLAG-MT1H were analyzed Western blotting.

**Table S1. Primer sequnces used in the current study.**

| **Primers for shRNA construction** | **Sequences (5'-3')** |
| --- | --- |
| MT1DP shRNA 1 F | CCGGAATGCAAAGAGTACAAATGCACTCGAGTGCATTTGTACTCTTTGCATTTTTTTG |
| MT1DP shRNA 1 R | AATTCAAAAAAATGCAAAGAGTACAAATGCACTCGAGTGCATTTGTACTCTTTGCATT |
| MT1DP shRNA 2 F | CCGGTCCAGTTGTAAATAACGCAACCTCGAGGTTGCGTTATTTACAACTGGATTTTTG |
| MT1DP shRNA 2 R | AATTCAAAAATCCAGTTGTAAATAACGCAACCTCGAGGTTGCGTTATTTACAACTGGA |
| **Primers for probe construction** | **Sequences (5'-3')** |
| MT1DP probe F | TAAGGCCAACAATGTTTATTATCA |
| MT1DP probe R | CCTGTGGCTTAGGAACTCCAGCCT |
| **Primers for plasmid construction** | **Sequences (5'-3')** |
| pGEMT-MT1DP F | CCTGTGGCTTAGGAACTCCAGCCTCACCTG |
| pGEMT-MT1DP R | TAAGGCCAACAATGTTTATTATC |
| MSP-MT1DP F | GCCGATATCATAGAACATCCCTGGGGCAGG |
| MSP-MT1DP R | TGCTCTAGAAAGGCCAACAATGTTTATTATCATTCC |
| MT1DP mutant F | GAAGAACTGCTGCTCATACAGCCCCATGGGCTG |
| MT1DP mutant R | CAGCCCATGGGGCTGTATGAGCAGCAGTTCTTC |
| MT1H C+3'UTR F | ATGGACCCCAACTGCTCCTGCGAG |
| MT1H C+3'UTR R | TCAAGTCTAAGTGTTTAATTATTATTCA |
| MT1H 3'UTR F | TGTCGGGACAGCCCTGCTGTCAGA |
| MT1H 3'UTR R | GTGTTTAATTATTATTCACATATTTCACAG |
| FLAG-MT1H CDS+3'UTR F | CCCAAGCTTATGGACCCCAACTGCTCCTGCGAG |
| FLAG-MT1H CDS+3'UTR R | CGGGGTACCTCAAGTCTAAGTGTTTAATTATTATTCA |
| pGL3-MT1H 3'UTR F | CTGTCGGGACAGCCCTGCTGTCAGATGAAAACAGAATGACACGTAAAC |
| pGL3-MT1H 3'UTR R | CGAGTTTACGTGTCATTCTGTTTTCATCTGACAGCAGGGCTGTCCCGACAGAGCT |
| pGL3-MT1H 3'UTR mutant F | CTGTCGGGACAGCCATACAGTCAGATGAAAACAGAATGACACGTAAAC |
| pGL3-MT1H 3'UTR mutant R | TCGAGTTTACGTGTCATTCTGTTTTCATCTGACTGTATGGCTGTCCCGACAGAGCT |
| pGL3-MT1DP 3’UTR F | CCTCTTCCTAAAGTGGACTCCTTTGCTTTGCACTTCTCGAGCTTTCTCCC |
| pGL3-MT1DP 3’UTR F | TCGAGGGAGAAAGCTCGAGAAGTGCAAAGCAAAGGAGTCCACTTTAGGAAGAGGAGCT |
| pGL3-MT1DP 3’UTR mutant F | CCTCTTCCTAAAGTGGACTCCTTTGCTTTCCAGTTCTCGAGCTTTCTCCC |
| pGL3-MT1DP 3’UTR mutant F | TCGAGGGAGAAAGCTCGAGAACTGGAAAGCAAAGGAGTCCACTTTAGGAAGAGGAGCT |
| **Primers for PCR** | **Sequences (5'-3')** |
| LET F | GTTGTTGTTGCATTGGGGT |
| LET R | AAGATGGAGAGTGGAGCCT |
| HEIH F | CCTCTTGTGCCCCTTTCTT |
| HEIH R | ATGGCTTCTCGCATCCTAT |
| MEG3 F | CTGCCCATCTACACCTCACG |
| MEG3 R | CTCTCCGCCGTCTGCGCTAGGGGCT |
| H19 F | ATCGGTGCCTCAGCGTTCGG |
| H19 R | CTGTCCTCGCCGTCACACCG |
| HULC F | ACCTCCAGAACTGTGATCCAAAATG |
| HULC R | TCTTGCTTGATGCTTTGGTCTG |
| MVIH F | AATTTTGCACATCTGAACAGCC |
| MVIH R | TTCAAAATCCCACTACGCCCA |
| DREH F | GCTAACGAACAAAGCCAGA |
| DREH R | CCCTATTCTCATGCAAGGA |
| PCNA-AS1 F | GTCCTTGAGTGCCTCCAACAC |
| PCNA-AS1 R | ACCAGCTAGACTTTCCTCCTTCC |
| PVT1 F | GCCCCTTCTATGGGAATCACTA |
| PVT1R | GGGGCAGAGATGAAATCGTAAT |
| UCA1F | TTTGCCAGCCTCAGCTTAAT |
| UCA1 R | TTGTCCCCATTTTCCATCAT |
| RoR F | AGGAAGCCTGAGAGTTGGC |
| RoR R | CTCAGTGGGGAAGACTCCAG |
| LALR1 F | ACGGGTGCGGGTTTAGG |
| LALR1 R | TCCAGGGCCGACTCCAT |
| VLDLR F | AGCAGTCACATTCATCGCAC |
| VLDLR R | GAGGAATAGGTGCGAACTGC |
| GAS5 F | CTTGCCTGGACCAGCTTAAT |
| GAS5 R | CAAGCCGACTCTCCATACCT |
| PTENP1F | AAGTAAGGACCAGAGACAAAAAGG |
| PTENP1R | TGCCACTGGTCTATAATCCACA |
| TUG1F | CCAGACCCTCAGTGCAAACT |
| TUG 1R | AATCAGGAGGCACAGGACA |
| ZFAS1 F | AGGCTTCATACGCTATTGTCCT |
| ZFAS1 R | GTGGTGACTCCCTCTTCCAA |
| SRA F | AGGATGGATCCCCCAGAGT |
| SRA R | TGGGAGCCTTACTTGAAGGAG |
| ANRIL F | TTGTGAAGCCCAAGTACTGC |
| ANRIL R | TTCACTGTGGAGACGTTGGT |
| CUDR F | GCACCCTAGACCCGAAA |
| CUDR R | GCCACCTGGACGGATAT |
| WT1-AS F | TAGTTAAGCAGATTCATAGA |
| WT1-AS R | GATGTCATAGTCCTCTTC |
| ZNFX1F | AAGCCACGTGCAGACATCTA |
| ZNFX1 R | CTACTTCCAACACCCGCATT |
| PANDAR F | CCCAACAAACAAGGGGTGG |
| PANDAR R | GTGGCCAAAGGATCTGACGA |
| HOTTIP F | CCTAAAGCCACGCTTCTTTG |
| HOTTIP R | TGCAGGCTGGAGATCCTAGT |
| HIF1A-AS1 F | GGTACTTTACGCACCCTGCT |
| HIF1A-AS1 R | GCTAGGGCAGGAAAGAGCAA |
| UFC1 F | TCCAACCTGAGTGACATAGCGA |
| UFC1 R | CTGACCTCCAACTCCAACGAAT |
| MT1DP F | TCAAGGCCAAAGGTGGCTCCTGCAC |
| MT1DP R | GCACGGCAGCTGCACTTCACCAATG |
| ATB F | TCTGGCTGAGGCTGGTTGAC |
| ATB R | ATCTCTGGGTGCTGGTGAAGG |
| URHC F | TGTTTATGTGAGAGGAGAAAGGAAG |
| URHC R | CACTAGAGGTCTGCAAATAAAGTGA |
| INXS F | CCCCCTCCAGGTACCAGAAC |
| INXS R | CCACTGGTGCTTTCGATTTGA |
| TUC339 F | GATGAGGCCCCGAGTTTAAT |
| TUC339 R | AGATGGAGGATCGGTGTGAA |
| PANDAR F | CCCAACAAACAAGGGGTGG |
| PANDAR R | GTGGCCAAAGGATCTGACGA |
| ENST00000563280 F | TTCATCGGCTGCGTATTCG |
| ENST00000563280 R | TTGCCTTCTAGTCGCCTCC |
| MIR31AS F | TTCTGTCCTCCTACTCGGACCC |
| MIR31AS R | CCTCCAGAGTTTGGTTTTGTGTC |
| NEAT1 F | CTTCCTCCCTTTAACTTATCCATTCAC |
| NEAT1 R | CTCTTCCTCCACCATTACCAACAATAC |
| human GAPDH-F | GAAGGTGAAGGTCGGAGT |
| human GAPDH-R | GAAGATGGTGATGGGATTTC |
| CRNDE F | GCGGAGGAGAGGTGTTAAGTGT |
| CRNDE R | AACAGGTTTTACCTCCTTATCTTCAGAA |
| IRAIN F | CGACACATGGTCCAATCACTGTT |
| IRAIN R | AGACTCCCCTAGGACTGCCATCT |
| RUNXOR F | CCTGTTCACGGTCCAAACTGG |
| RUNXOR R | CGGCAAGATCACAGTCCCTAGC |
| CTGF F | CAGCATGGACGTTCGTCTG |
| CTGF R | AACCACGGTTTGGTCCTTGG |
| CYR61 F | ACCGCTCTGAAGGGGATCT |
| CYR61 R | ACTGATGTTTACAGTTGGGCTG |
| RhoC F | GGAGGTCTACGTCCCTACTGT |
| RhoC R | CGCAGTCGATCATAGTCTTCC |
| miR-214 loop | CTCAACTGGTGTCGTGGAGTCGGCAATTCAGTTGAGACTGCCTG |
| U6 sn F | CTCGCTTCGGCAGCACA |
| U6 sn R | AACGCTTCACGAATTTGCGT |
| miR-214 F | ACACTCCAGCTGGGACAGCAGGCACAGACA |
| miR-214 R | TGGTGTCGTGGAGTCG |
| MT1H F | TGGGAACTCCAGTCTCACCT |
| MT1H R | GTTTTCATCTGACAGCAGGGC |
| MT1E F | TCAGGTTGGGAGGGAACTCAA |
| MT1E R | GAAAGCCTGGAGAGGGAATGA |
| MT1A F | CTCGAAATGGACCCCAACT |
| MT1A R | ATATCTTCGAGCAGGGCTGTC |
| MT1G F | CTTCTCGCTTGGGAACTCTA |
| MT1G R | AGGGGTCAAGATTGTAGCAAA |
| MT1B F | GCTTGTCTTGGCTCCACA |
| MT1B R | AGCAAACCGGTCAGGTAGTTA |
| MT1F F | AGTCTCTCCTCGGCTTGC |
| MT1F R | ACATCTGGGAGAAAGGTTGTC |
| MT1X F | TCTCCTTGCCTCGAAATGG |
| MT1X R | CACAGCTGTCCTGGCATCA |
| MT1M F | GCTTGAGATCTCCAGCCTTACC |
| MT1M R | TTGCAGGAGGTGCATTTG |
| MT1L F | TCGCCTCTCCCGTCATTT |
| MT1L R | AGCAGGGCTGTCCCCA |
| MT1JP F | CGCTCCACCACGCCGTCCACG |
| MT1JP R | ACTTGCAGGAGCCGGCGCACGT |
| MT1CP F | ATGGACCTCAACTGCTCCTG |
| MT1CP R | AGCAGGAGCAGCCTCTTCTTGC |
| MT1IP F | GCTCCTGCTCCACCTCCTGCAAAT |
| MT1IP R | CTACATCAGGAGCAGCAGCTGC |
| MT3 F | CGACAACGGCTCCGGCATGT |
| MT3 R | TGCCGTGCTCGATGGGGTACT |

**Table S2. Mass spectrometry-identified proteins.**

| **Number** | **Protein Name** |
| --- | --- |
| 1 | ELAV-like protein 1 |
| 2 | Ras-related protein Rab-6B |
| 3 | Ras-related protein Rab-4B |
| 4 | Ras-related protein Rab-7a |
| 5 | Ras-related protein Rap-1b-like protein |
| 6 | Rho-related GTP-binding protein RhoG |
| 7 | Rho-related GTP-binding protein RhoC |
| 8 | SWI/SNF complex subunit SMARCC1 |
| 9 | Hepatocyte growth factor receptor |
| 10 | Tyrosine-protein kinase Lyn |
| 11 | Tyrosine-protein kinase Yes |
| 12 | Cyclin-dependent kinase 3 |
| 13 | Fibroblast growth factor receptor 4 |
| 14 | Cyclin-dependent kinase 13 |
| 15 | DNA damage-binding protein 2 |
| 16 | E3 ubiquitin-protein ligase TRAF7 |
| 17 | GTP-binding protein SAR1a |
| 18 | Inhibitor of nuclear factor kappa-B kinase subunit alpha |
| 19 | Platelet-activating factor acetylhydrolase IB subunit gamma |

**Table S3. Thirty-two candidate genes from RNA-Seq data for further analyses.**

|  | Gene Symbol | Scrambled shRNA untreated | Scrambled shRNA upon Cd 20 μM | MT1DP shRNA untreated | MT1DP shRNA upon Cd at 20 μM |
| --- | --- | --- | --- | --- | --- |
| 1 | FGF11 | 1.02 | 2.384615385 | 0.482758621 | 0.6261682 |
| 2 | ZNF488 | 2.45 | 0.701923077 | 6.502463054 | 3.4205607 |
| 3 | JUN | 243.04 | 101.8076923 | 57.91133005 | 66.859813 |
| 4 | STAT5B | 34.96 | 16.25961538 | 34.63054187 | 31.785047 |
| 5 | NFATC1 | 0 | 3.817307692 | 0.068965517 | 0.6074766 |
| 6 | SMAD3 | 6.19 | 2.951923077 | 9.733990148 | 6.9719626 |
| 7 | TGFB1 | 4.8 | 6.019230769 | 4.798029557 | 3.8411215 |
| 8 | RRAS | 30.49 | 7.961538462 | 28.37438424 | 19.009346 |
| 9 | DTX4 | 4.33 | 3.019230769 | 18.22660099 | 10.17757 |
| 10 | SYT12 | 11.55 | 4.596153846 | 22.98522167 | 10.654206 |
| 11 | TSC1 | 3.09 | 15.45192308 | 3.645320197 | 6.3831776 |
| 12 | PIK3C2B | 6.63 | 4.913461538 | 16.72906404 | 16.504673 |
| 13 | TGM2 | 6.24 | 6.817307692 | 2.374384236 | 2.8224299 |
| 14 | TGFBR1 | 61.51 | 26.125 | 14.62068966 | 12.345794 |
| 15 | FGF2 | 24.03 | 19.01923077 | 8.532019704 | 8.9252336 |
| 16 | NR4A1 | 86.03 | 127.875 | 27.5270936 | 47.373832 |
| 17 | ATOH8 | 0.89 | 0.288461538 | 2.02955665 | 1.411215 |
| 18 | ITGA5 | 129.11 | 135.7211538 | 41.60591133 | 59.018692 |
| 19 | CADM4 | 3.55 | 3.769230769 | 8.147783251 | 13.168224 |
| 20 | DUSP9 | 2.55 | 1.105769231 | 8.049261084 | 9.7850467 |
| 21 | ERBB2 | 5.8 | 4.125 | 12.94581281 | 8.728972 |
| 22 | DENND4A | 3.52 | 1.413461538 | 5.18226601 | 3.6635514 |
| 23 | EGFL8 | 0 | 0 | 1.684729064 | 3.3551402 |
| 24 | CDKN1A | 78.8 | 103.5961538 | 39.7635468 | 35.64486 |
| 25 | MYCT1 | 0 | 4.644230769 | 0 | 0.5981308 |
| 26 | CCN1 | 37.18 | 111.1923077 | 27.8226601 | 41.691589 |
| 27 | CCN2 | 9.82 | 63.95192308 | 7.024630542 | 18.093458 |
| 28 | PRKCB | 0.15 | 0.153846154 | 1.802955665 | 0.8504673 |
| 29 | KITLG | 2.81 | 2.701923077 | 8.807881773 | 6.1962617 |
| 30 | CCNG2 | 1.53 | 2.307692308 | 4.492610837 | 4.5233645 |
| 31 | PTK2B | 4.47 | 2.432692308 | 10.70935961 | 5.9252336 |
| 32 | TGFBR1 | 61.51 | 26.125 | 14.62068966 | 12.345794 |
